# Method of Higher-order Operators for Quantum Optomechanics

**DOI:** 10.1038/s41598-018-30068-7

**Published:** 2018-08-01

**Authors:** Sina Khorasani

**Affiliations:** Vienna Center for Quantum Science and Technology, Boltzmanngasse 5, 1090 Vienna, Austria

## Abstract

We demonstrate application of the method of higher-order operators to nonlinear standard optomechanics. It is shown that a symmetry breaking in frequency shifts exists, corresponding to inequivalency of red and blue side-bands. This arises from nonlinear higher-order processes leading to inequal detunings. Similarly, a higher-order resonance shift exists appearing as changes in both of the optical and mechanical resonances. We provide the first known method to explicitly estimate the population of coherent phonons. We also calculate corrections to spring effect due to higher-order interactions and coherent phonons, and show that these corrections can be quite significant in measurement of single-photon optomechanical interaction rate. It is shown that there exists non-unique and various choices for the higher-order operators to solve the optomechanical interaction with different multiplicative noise terms, among which a minimal basis offers exactly linear Langevin equations, while decoupling one Langevin equation and thus leaving the whole standard optomechanical problem exactly solvable by explicit expressions. We finally present a detailed treatment of multiplicative noise as well as nonlinear dynamic stability phases by the method of higher-order operators. Similar approach can be used outside the domain of standard optomechanics to quadratic and all other types of nonlinear interactions in quantum physics.

## Introduction

Nonlinear quantum interactions with stochastic noise input stand among the most difficult analytical challenges to solve in the context of stochastic differential equations. While linearized interactions remain accurate for description of many experiments, a certain class of quadratic and higher-order physical phenomena cannot be normally understood under linearized approximations. While in classical problems the resulting Langevin equations are scalar functions, in quantum problems one has to deal with nonlinear operator differential equations. If expanded unto base kets, bosonic operators can assume infinite-dimensional matrix forms, rendering the solution entirely intractable.

Such classes of nonlinear operator problems can be addressed by construction of Fokker-Planck or nonlinear Schrödinger equations, among which there exists a one-to-one correspondence. The Fokker-Planck equation^1–5^ is actually equivalent to the nonlinear Schrödinger equation with bosonic operator algebra, and its moments^6^ translate into nonlinear Langevin equations. The method of master equations^7,8^ also can be used in combination with the quasi-probablity Wigner functions^9,10^ to deal with nonlinear quantum interactions. The master equation approach is reasonably accurate as long as Born and Markov approximations are not employed^11^. But none of these methods is probably as convenient as the method of Langevin equations^12–15^, which has found popularity in the context of quantum optoemchanics^16–29^.

Being an inherently nonlinear interaction among photonic and phononic baths^30–38^, the standard quantum optomechanics is normally described by linearized Langevin equations^12–15^. This will suffice to address a majority of complex experimental situations such as optomechanical-induced transparency^39–41^ and polaron anti-crossing^42^, but effects such as non-classical states of light^9,10,43,44^, optomechanical emission of real photons from vacuum^45^, photon blockade^43^, nonlinear self-oscillations^46–50^, and chaos^51,52^ are all among manifestations of nonlinear regimes in standard optomechanics, which need description using nonlinear algebra. Also, biquadratic interactions (mostly referred to as quadratic interactions) among bosonic baths remain a hurdle. In quadratic optomechanics^53–64^, which is a topic of growing interest in the recent year, having an analytical tool capable of addressing such kinds of nonlinearity is advantageous. A perturbation technique based on the expansion of time-evolution operators^64^ is employed to investigate quadratic interactions and it has been shown that for mechanical frequencies exceeding optical frequencies a new unexplored regime appears in which the roles of optical and mechanical partitions are interchanged.

Recently, the author has reconsidered the theoretical description of optomechanics^65^ and shown that quadratic interactions are subject to two corrections resulting from momentum conservation and relativistic effects. Such types of quadratic corrections become significant when the mechanical frequency is within the same order of or exceeds electromagnetic frequency. Furthermore, an analytical approach is proposed to tackle nonlinear quantum interactions^66^ and a method of expansion unto higher-order operators is proposed and investigated in details.

In this article, the higher-order operator approach recently proposed by the author^66–69^ is employed to address the standard optomechanics, and it is shown that there exists a minimal choice of higher-order operator basis which leads to exactly linear and fully separable Langevin equations with multiplicative input noise terms^70^. We also present a full mathematical treatment of multiplicative noise terms, which turn out to play a crucial rule in higher-order quantum optomechanics. This allows one to provide an exact and explicit solution using an operator-based method to solve the optomechanical interactions in the nonlinear regime. There exists higher-order effects appearing at high optical pump rates, and can be predicted using the method discussed here. These include inequivalent red and blue detunings, higher-order resonance shift and spring effects, and also zero-point-field induced optomechanical shift of mechanical frequency. The inequivalency of red and blue detuned side-bands, which appears as a counter-intuitive difference in their respective frequency shifts, is different from the well-known anomalous Stokes-Anti-Stokes symmetry breaking^71–73^ which is connected to different scattering amplitudes. The same method of higher-order operator algebra has been recently used independently as well^74^.

We also show for the first time that the introduced method of higher-order operators can be used to estimate the coherent population of phonons in the optomechanical cavity, here referred to as the coherent phonon number. This quantity can not only be calculated explicitly in terms of optomechanical parameters, but also, can be found by fitting the expressions of corrected spring effect to the experimental observations. Also, dynamic linear and nonlinear stability phases in red and blue-detuned drives can be well computed and estimated using the method of higher-order operators.

## Results

The standard optomechanical Hamiltonian reads^16–20^

1 $${{\mathbb{H}}}_{{\rm{OM}}}=\hslash {\rm{\Omega }}\hat{m}-\hslash {\rm{\Delta }}\hat{n}-\hslash {g}_{0}\hat{n}(\hat{b}+{\hat{b}}^{\dagger }),$$

where $\hat{n}={\hat{a}}^{\dagger }\hat{a}$ and $\hat{m}={\hat{b}}^{\dagger }\hat{b}$ are photon and phonon number operators with $\hat{a}$ and $\hat{b}$ respectively being the photon and phonon annihilators, Ω is the mechanical frequency, Δ is optical detuning from cavity resonance, and *g*_0_ is the single-photon optomechanical interaction rate. The interaction ${{\mathbb{H}}}_{{\rm{OM}}}$ is not quadratic, but is still cubic nonlinear. It is normally solved by a straightforward linearization^18–20^, but can be also solved at the second-order accuracy using the higher-order operators described in the preceding article^66^.

In order to form a closed basis of operators, we may choose either the higher-order operators

2 $${\{A\}}^{{\rm{T}}}=\{\hat{a},\hat{a}\hat{b},\hat{a}{\hat{b}}^{\dagger }\},$$

of the second-degree, which forms a 3 × 3 system of Langevin equations, or

3 $${\{A\}}^{{\rm{T}}}=\{\hat{a},\hat{b},\hat{a}\hat{b},\hat{a}{\hat{b}}^{\dagger },\hat{n},\hat{c}\},$$

which forms a 6 × 6 system of Langevin equations. Here, we adopt the definition $\hat{c}=\frac{1}{2}{\hat{a}}^{2}$^65,66^.

It is easy to verify that this system is exactly closed, by calculation of all possible commutation pairs between the elements. Out of the 6! commutators, the non-zero ones are $[\hat{a},\hat{n}]=-\,[\hat{a}{\hat{b}}^{\dagger },\hat{b}]=\hat{a}$, $[\hat{a}\hat{b},\hat{n}]=\hat{a}\hat{b}$, $[\hat{a}{\hat{b}}^{\dagger },\hat{n}]=\hat{a}{\hat{b}}^{\dagger }$, and $[\hat{a}\hat{b},\hat{a}{\hat{b}}^{\dagger }]=[\hat{c},\hat{n}]=2\hat{c}$, which is obviously a closed basis. Now, one may proceed with composition of the Langevin equations.

The applicability of the basis (2) becomes readily clear by calculating the braket $[\hat{a},{{\mathbb{H}}}_{{\rm{OM}}}]$ as appears in the corresponding Langevin equation. The terms involving the second-degree operators $\hat{a}\hat{b}$ and $\hat{a}{\hat{b}}^{\dagger }$ immediately show up. The key in the method of higher-order operators is to keep these operator pairs, triplets and so on together, as each combination has a clear corresponding physical process. While $\hat{a}$ and $\hat{b}$ refer to individual ladder operators, $\hat{a}\hat{b}$ and $\hat{a}{\hat{b}}^{\dagger }$ respectively construct the blue and red 1-photon/1-phonon processes. For this reason, it is probably more appropriate to call these higher-degree operator combinations as processes.

The Langevin equations for the blue $\hat{a}\hat{b}$ and red $\hat{a}{\hat{b}}^{\dagger }$ processes do not close on themselves, because of the appearance of third-order blue- and red-like processes $\hat{a}{\hat{b}}^{2}$ and $\hat{a}{\hat{b}}^{\dagger 2}$, describing 1-photon/2-phonon processes. Similarly, every *j*-th order blue- or red-like process such as $\hat{a}{\hat{b}}^{j}$ and $\hat{a}{\hat{b}}^{\dagger j}$ will lead to the *j* + 1-order process. Hence, the infinite-dimensional basis $\{\hat{a}\}\cup \{\forall \hat{a}{\hat{b}}^{j},\hat{a}{\hat{b}}^{\dagger j};j\in {\mathscr{N}}\,\}$ can provide an exact solution to the optomechanics. Furthermore, the convergence of solutions basis on such expansions would be questionable when *g*_0_ ≪ Ω is violated. In general, the *j*-th order processes correspond to the 1-photon/*j*-phonon interactions and contribute to the *j* + 1-order sidebands. In this article, it has been shown that under practical conditions, it is unnecessary to take account of the processes *j* ≥ 2 and the 3 × 3 basis (2) is rather sufficient for most practical purposes. Nonetheless, *j* = 2 processes contribute significantly to nonlinear stability and second-order mechanical sidebands. While the use of an infinite-dimensional basis is surprisingly unnecessary in still a higher-order formulation, using the compact minimal basis to be discussed in the following can lead to the mathematically exact solution. The choice of basis is not unique, and every non-degenerate linear combination of bases leads to another equivalent form. One may for instance arbitrate the three-dimensional linear basis ${\{A\}}^{{\rm{T}}}=\{\hat{a},\hat{b},{\hat{b}}^{\dagger }\}$ or the four-dimensional linear basis ${\{A\}}^{{\rm{T}}}=\{\hat{a},\hat{b},{\hat{a}}^{\dagger },{\hat{b}}^{\dagger }\}$ as is taken in the context of linearized standard optomechanics^18–20^, the five-dimensional all-Hermitian basis ${\{A\}}^{{\rm{T}}}=\{\hat{n},\hat{m},{\hat{n}}^{2},\hat{n}(\hat{b}+{\hat{b}}^{\dagger }),i\hat{n}(\hat{b}-{\hat{b}}^{\dagger })\}$^64^, and ultimately the minimal three-dimensional basis

4 $${\{A\}}^{{\rm{T}}}=\{{\hat{n}}^{2},\hat{n}\hat{b},\hat{n}{\hat{b}}^{\dagger }\}=\{\hat{N},\hat{B},{\hat{B}}^{\dagger }\},$$

assumed here, which is of the fourth-degree. We shall later observe that while (3) is necessary to construct the closed Langevin equations, a second-order linearization will be needed to decouple three operators, leaving only the basis (2) in effect. Quite remarkably, however, and in a similar manner, the use of minimal basis (4) turns out to be fairly convenient to construct the optomechanical Langevin equations. This is not only since the Langevin equations take on exactly linear forms, but also eventually the equation for $\hat{N}$ and ${\hat{B}}^{\dagger }$ will decouple. This leaves the whole standard optomechanical interaction exactly solvable through integration of only one linear differential equation in terms of $\hat{B}$. The main difference between using various choices of higher-order operator bases^66^ is the noise terms. It turns out that the definition and higher-order operators lead to multiplicative noise inputs, which once known, the problem will be conveniently solvable. Full mathematical treatment of multiplicative noise terms is necessary for description of some various phenomena and this will be discussed in §S10 of supplementary information.

### Side-band Inequivalence

Defining Δ_*b*_ and Δ_*r*_ respectively as the blue and red frequency shifts of sidebands, it is possible to show that these two quantities do not necessarily agree in magnitude, such that Δ_*b*_ + Δ_*r*_ ≠ 0. As shown in §S4 of supplementary information, an explicit relation for the side-band inequivalence $\delta {\rm{\Delta }}=\frac{1}{2}({{\rm{\Delta }}}_{{\rm{r}}}+{{\rm{\Delta }}}_{{\rm{b}}})$ can be found through series expansion of the eigenvalues of the coefficient matrix from (S19). With some algebra, it is possible to show that for *g*_0_ ≪ Ω correct to the fourth-order, we get

5 $$\frac{\delta {\rm{\Delta }}}{{\rm{\Omega }}}\approx {(\frac{{g}_{0}}{{\rm{\Omega }}})}^{2}(\bar{n}+\frac{1}{2})-2{(\frac{{g}_{0}}{{\rm{\Omega }}})}^{4}(\bar{n}+\frac{1}{2})(\bar{m}+\frac{1}{2})\mathrm{.}$$

**Figure 1:**
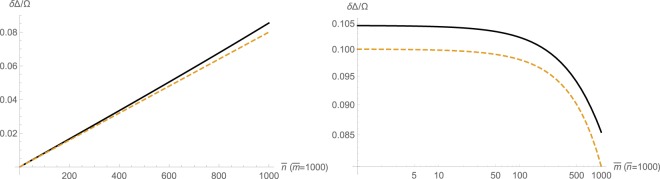
Normalized inequivalence *δ*Δ/Ω = (Δ_b_ + Δ_r_)/2Ω of sideband frequency detunings Δ_r_ and Δ_b_ versus intracavity photon $\bar{n}$ and coherent phonon $\bar{m}$ for *g*_0_/Ω = 10^−3^. Solid lines are from exact numerical calculations and dashed lines are from the asymptotic expansion (5).

Here, $\bar{n}({\rm{\Delta }})$ is the intracavity photon population and $\bar{m}({\rm{\Delta }})$ is the coherent phonon population given by

6 $$\bar{m}({\rm{\Delta }})\approx \frac{32{g}_{0}^{2}{{\rm{\Omega }}}^{2}({\gamma }^{2}+\gamma {\rm{\Gamma }}+4{{\rm{\Delta }}}^{2})}{({\gamma }^{2}+4{{\rm{\Delta }}}^{2}){({{\rm{\Gamma }}}^{2}+4{{\rm{\Omega }}}^{2})}^{2}}{\bar{n}}^{2}({\rm{\Delta }})={g}_{0}^{2}\zeta ({\rm{\Delta }}){\bar{n}}^{2}({\rm{\Delta }}),$$

where Γ is the mechanical decay rate, and *γ* = *κ* + Γ is the total optomechanical decay rate with *κ* being the optical decay rate, as proved in details in §S6 of supplementary information using the method of higher-order operators. Also, $\bar{n}({\rm{\Delta }})$ can be found from numerical solution of a third-order algebraic equation (S9). The relationship $\bar{m}\propto {\bar{n}}^{2}$ signifies the fact that mechanical oscillations are nonlinearly driven by optical radiation pressure. A typical behavior of this phenomenon is illustrated in Fig. 1.

There is a related polaritonic splitting effect^39^, as a result of anti-crossing between the optomechanically interacting optical and mechanical resonances generated across either of the mechanical side-bands, amount of which happens to be exactly 2*δ*Δ. This has nothing to do with the side-band asymmetry, which happens to occur on the two opposite sides of the main cavity resonance. It should be mentioned that observation of this phenomenon in superconducting electromechanics^75^ as well as parametrically actuated nano-string resonators^76,77^ can potentially yield the most clear results due to various experimental conditions. In fact, intracavity photon numbers as large as 10^6^ and 10^8^ and more are attainable respectively in superconducting electromechanics and optically-trapped nano-particle optomechanics.

A close inspection of a very high-resolution measurement on a side-band resolved microtoroidal disk^78^ yields a side-band inequivalence of *δ*Δ ≈ 2*π* × (142 ± 36) Hz, which perfectly complies to (5) if $\bar{n}$ = (5.1 ± 1.3) × 10^3^. Unfortunately, further such a high resolution measurements on deeply side-band resolved optomechanical cavities are not reported elsewhere to the best knowledge of authors. Nevertheless, clear signatures of side-band inequivalence can be easily verified in few other experiments^75,76,79^. Remarkably, recent measurements on Stokes-Anti-Stokes scattering from multi-layered MoTe_2_ exhibits a difference in frequency shift as large as 7% for the five-layered sample^72^, which corresponds to 0.88 ± 0.11 cm^−1^.

Also, a recent landmark experiment on room-temperature quantum optomechanical correlations^80^ has reported measurements which coincidentally exhibit a sideband inequivalence up to 4 kHz and roughly agree to the approximation $\delta {\rm{\Delta }}\approx {g}_{0}^{2}\bar{n}/{\rm{\Omega }}$. This issue remains, nevertheless, as an open problem in the context of experimental quantum optomechanics.

In any experimental attempt to measure this phenomenon, a side-band resolved cavity could be driven on resonance and noise spectra of the two mechanical side bands be measured with extreme precision in a heterodyne setup. Even in case of well-known thermo-optical effects and two-photon dispersion or absorption which cause drifts in the optical resonance and other optomechanical parameters^81^, this effect should be still observable in principle. The reason is that the amount of inequivalence is actually independent of the exact pump frequency as long as intracavity photon population does not change significantly. So, it should be sufficient only if the cavity is driven on or close to the optical resonance for the side-bands to be sufficiently different in their frequency shifts.

### Higher-order Resonance Shift

The contribution of the off-diagonal terms to the mechanical frequency Ω in the coefficients matrix of optomechanical Langevin equations (S20) of supplementary information, can be ultimately held responsible for the so-called optomechanical spring effect^18–20,61,82–86^. As the result of optomechanical interaction, both of the optical and mechanical resonance frequencies and damping rates undergo shifts. Even at the limit of zero input optical power *α* = 0 and therefore zero cavity photon number $\bar{n}$ = 0, it is possible to show that there is a temperature-dependent shift in the mechanical resonance frequency, markedly different from the lattice-expansion dependent effect. This effect is solely due to the optomechanical interaction with virtual cavity photons, which completely vanishes when *g*_0_ = 0. In close relationship to the shift of resonances, we can also study the optomechanical spring effect with the corrections from higher-order interactions included.

The analysis of spring effect is normally done by consideration of the effective optomechanical force acting upon the damped mechanical oscillator, thus obtaining a shift in squared mechanical frequency *δ*(Ω^2^), whose real and imaginary parts give expressions for *δ*Ω and *δ*Γ. Corrections to these two terms due to higher-order interactions are discussed in §. Here, we demonstrate that the analysis using higher-order operator algebra can recover some important lost information regarding the optical and mechanical resonances when the analysis is done on the linearized basis ${\{A\}}^{{\rm{T}}}=\{\hat{a},{\hat{a}}^{\dagger },\hat{b},{\hat{b}}^{\dagger }\}$.

To proceed, we consider finding eigenvalues of the matrix **M** as defined in (S19) of supplementary information. Ignoring all higher-order nonlinear effects beyond the basis ${\{A\}}^{{\rm{T}}}=\{\hat{a},\hat{a}\hat{b},\hat{a}{\hat{b}}^{\dagger }\}$, we set *s* = 0. This enables us to search for the eigenvalues of the coefficients matrix **M** as

7 $${\rm{eig}}[{\bf{M}}]={\rm{eig}}\,[\begin{array}{ccc}i{\rm{\Delta }}-\frac{\kappa }{2} & i{g}_{0} & i{g}_{0}\\ i(G+{f}^{+}) & -i({\rm{\Omega }}-{\rm{\Delta }})-\frac{\gamma }{2} & 0\\ -i(G-{f}^{-}) & 0 & i({\rm{\Omega }}+{\rm{\Delta }})-\frac{\gamma }{2}\end{array}]=i\{\begin{array}{c}{\rm{\Delta }}+{\lambda }_{1}+i{\gamma }_{1}\\ {\rm{\Delta }}+{\lambda }_{2}+i{\gamma }_{2}\\ {\rm{\Delta }}+{\lambda }_{3}+i{\gamma }_{3}\end{array}\}=i\{\begin{array}{c}{\rm{\Delta }}+{\eta }_{1}({\rm{\Delta }},T)\\ {\rm{\Delta }}+{\eta }_{2}({\rm{\Delta }},T)\\ {\rm{\Delta }}+{\eta }_{3}({\rm{\Delta }},T)\end{array}\},$$

in which *G* = *g*_0_$\bar{n}$, ${f}^{\pm }={g}_{0}(\bar{m}+\frac{1}{2})\pm \frac{1}{2}{g}_{0}$, ${\lambda }_{j}=\Re [{\eta }_{j}]$ and *γ*_*j*_ = ℑ[*η*_*j*_] with *j* = 1, 2, 3 are real valued functions of Δ and bath temperature *T*. The temperature *T* determines $\bar{m}$ while $\bar{n}$ is a function of Δ as well as input photon rate *α*. In general, the three eigenvalues *η*_*j*_ = *λ*_*j*_(Δ, *T*) + *iγ*_*j*_(Δ, *T*), *j* = 1, 2, 3 are expected to be deviate from the three free-running values ${\psi }_{1}=i\frac{1}{2}\kappa$, ${\psi }_{2}=-\,{\rm{\Omega }}+i\frac{1}{2}\gamma$, and ${\psi }_{3}={\rm{\Omega }}+i\frac{1}{2}\gamma$, as *η*_*j*_ ≈ *ψ*_*j*_ − Δ because of non-zero *g*_0_. Solving the three equations therefore gives the values of shifted optical and mechanical frequencies and their damping rates compared to the bare values in absence of optomechanical interactions with *g*_0_ = 0, given by $\delta {\rm{\Omega }}=-\,\frac{1}{2}\Re [{\eta }_{2}-{\eta }_{3}]-{\rm{\Omega }}$, $\delta \omega =-\,\frac{1}{2}\Re [{\eta }_{2}+{\eta }_{3}]$, *δ*Γ = ℑ[−2*η*_1_ + *η*_2_ + *η*_3_] − Γ, and *δ* = 2ℑ[*η*_1_] − *κ*. This method to calculate the alteration of resonances, does not regard the strength of the optomechanical interaction or any of the damping rates. In contrast, the known methods to analyze this phenomenon normally require *g* *κ* and Γ + *δ*Γ ≪ *κ*^19^.

### Corrections to Spring Effect

As shown in §S7 of supplementary information, the full expression for corrected spring effect is given as Put together combined, we get

8 $$\begin{array}{rcl}\delta {\rm{\Omega }}(w,{\rm{\Delta }}) & = & \frac{{g}_{0}^{2}\bar{n}{\rm{\Omega }}}{w}[\frac{{\rm{\Delta }}+w}{{({\rm{\Delta }}+w)}^{2}+\frac{1}{4}{\kappa }^{2}}+\frac{{\rm{\Delta }}-w}{{({\rm{\Delta }}-w)}^{2}+\frac{1}{4}{\kappa }^{2}}]\\  &  & +\,\frac{{g}_{0}^{2}\Re [\mu (w)]{\rm{\Omega }}}{w}[\frac{{\rm{\Delta }}+w}{{({\rm{\Delta }}+w)}^{2}+\frac{1}{4}{\kappa }^{2}}+\frac{{\rm{\Delta }}-w}{{({\rm{\Delta }}-w)}^{2}+\frac{1}{4}{\kappa }^{2}}]\\  &  & +\,\frac{{g}_{0}^{2}\Im [\mu (w)]{\rm{\Omega }}}{w}[\frac{\kappa }{{({\rm{\Delta }}+w)}^{2}+\frac{1}{4}{\kappa }^{2}}-\frac{\kappa }{{({\rm{\Delta }}-w)}^{2}+\frac{1}{4}{\kappa }^{2}}],\end{array}$$

9 $$\begin{array}{rcl}\delta {\rm{\Gamma }}(w,{\rm{\Delta }}) & = & \frac{{g}_{0}^{2}\bar{n}{\rm{\Omega }}}{w}[\frac{\kappa }{{({\rm{\Delta }}+w)}^{2}+\frac{1}{4}{\kappa }^{2}}-\frac{\kappa }{{({\rm{\Delta }}-w)}^{2}+\frac{1}{4}{\kappa }^{2}}]\\  &  & +\,\frac{{g}_{0}^{2}\Re [\mu (w)]{\rm{\Omega }}}{w}[\frac{\kappa }{{({\rm{\Delta }}+w)}^{2}+\frac{1}{4}{\kappa }^{2}}-\frac{\kappa }{{({\rm{\Delta }}-w)}^{2}+\frac{1}{4}{\kappa }^{2}}]\\  &  & -\,\frac{{g}_{0}^{2}\Im [\mu (w)]{\rm{\Omega }}}{w}[\frac{{\rm{\Delta }}+w}{{({\rm{\Delta }}+w)}^{2}+\frac{1}{4}{\kappa }^{2}}+\frac{{\rm{\Delta }}-w}{{({\rm{\Delta }}-w)}^{2}+\frac{1}{4}{\kappa }^{2}}]\mathrm{.}\end{array}$$

Here, the second and third terms on the rights hand sides of both equations are corrections to the spring effect due to the higher-order interactions, resulting from the temperature-dependent expressions

10 $$\begin{array}{rcl}\Re [\mu (w)] & = & \frac{w}{{\rm{\Omega }}}(\bar{m}+\frac{1}{2})+\frac{1}{2},\\ \Im [\mu (w)] & = & \frac{{\rm{\Gamma }}}{2{\rm{\Omega }}}(\bar{m}+\frac{1}{2})\mathrm{.}\end{array}$$

The temperature-dependence of (10) causes dependence of the spring effect on temperature as well. The influence of additional terms in (8) due to higher-order interactions can strongly influence any measurement of *g*_0_ through spring effect, as most easily can be observable in the weak coupling limit for Doppler cavities.

### Weak Coupling Limit

In the weakly coupled operation mode and far Doppler regime where *g*_0_ ≪ Ω and *κ* ≫ Ω ≫ Γ hold^87,88^, using (S9) of supplementary information with $\bar{n}\approx {({{\rm{\Delta }}}^{2}+\frac{1}{4}{\kappa }^{2})}^{-1}|\alpha {|}^{2}$, the spring equations are obtained from (8) by setting *w* = Ω as

11 $$\delta {\rm{\Omega }}({\rm{\Omega }},{\rm{\Delta }})\approx 2{\rm{\Delta }}{g}_{0}^{2}\frac{\bar{n}({\rm{\Delta }})+\bar{m}({\rm{\Delta }})+1}{{{\rm{\Delta }}}^{2}+\frac{1}{4}{\kappa }^{2}}\approx {g}_{0}^{2}[\frac{2{\rm{\Delta }}|\alpha {|}^{2}}{{({{\rm{\Delta }}}^{2}+\frac{1}{4}{\kappa }^{2})}^{2}}]+{g}_{0}^{4}[\frac{2{\rm{\Delta }}\zeta ({\rm{\Delta }})|\alpha {|}^{4}}{{({{\rm{\Delta }}}^{2}+\frac{1}{4}{\kappa }^{2})}^{3}}]\mathrm{.}$$

Here, |*α*| is photon input rate to the cavity with *α* being complex drive amplitude, and $\Re [\mu ({\rm{\Omega }})]=\bar{m}+1$ and ℑ[*μ*(Ω)] ≈ 0 from (10). The importance of this equation is that the optical spring effect is actually proportional to $\delta {\rm{\Omega }}\propto {g}_{0}^{2}(\bar{n}+\bar{m})\propto {g}_{0}^{2}\bar{n}\mathrm{(1}+{g}_{0}^{2}\zeta \bar{n})$ where *ζ*(Δ) is already defined in (S30). This shows that if *g*_0_ is to be determined from experimental measurement of the optical spring effect, then the experiment should be done at the lowest optical power possible, otherwise the term $\bar{m}\propto {g}_{0}^{2}{\bar{n}}^{2}$ becomes large and would result in an apparent change in *g*_0_. This fact also can explain why the measured *g*_0_ through optical spring effect using uncorrected standard expressions (S42) is always different from the design value, which could be attributed to the absence of the second term proportional to ${g}_{0}^{4}$ in the corrected optical spring effect using the higher-order algebra.

The above equation together with the fact that on the far red detuning Δ → +∞ we have $\bar{n}$(Δ) → 0, $\bar{m}$(Δ) → 0, and *δ*Ω(Ω, Δ) → 0, provides an alternate approximation for the resonant coherent phonon number $\bar{m}$(0) at zero-detuning as

12 $$\bar{m}\mathrm{(0)}\approx \frac{{\kappa }^{2}}{8{g}_{0}^{2}}{[\frac{\partial \delta {\rm{\Omega }}({\rm{\Omega }},{\rm{\Delta }})}{\partial {\rm{\Delta }}}]}_{{\rm{\Delta }}=0}-4\frac{|\alpha {|}^{2}}{{\kappa }^{2}}-1\approx \frac{32{g}_{0}^{2}{Q}_{{\rm{m}}}^{2}}{{{\rm{\Gamma }}}^{2}}{\bar{n}}^{2}\mathrm{(0)}\approx \frac{512{g}_{0}^{2}{Q}_{{\rm{m}}}^{2}}{{{\rm{\Gamma }}}^{2}{\kappa }^{4}}|\alpha {|}^{4},$$

where *Q*_m_ = Ω/Γ is the mechanical quality factor, and the expression within the brackets can be measured experimentally, and represents the slope of frequency displacement due to the spring effect versus detuning. The second expression proportional to ${\bar{n}}^{2}$(0) follows (S30) from §S6 of supplementary information where an explicit and accurate formula for $\bar{m}$(Δ) is found.

Noting |*α*|^2^ ∝ *P*_op_ reveals that while the intracavity photon number is propotional to the optical power as ${\bar{n}}^{2}$(0) ∝ *P*_op_, the coherent phonon population is proportional to the square of the optical power as $\bar{m}\mathrm{(0)}\propto {P}_{{\rm{op}}}^{2}$. This implies that the effects of coherent mechanical field gets important only at sufficiently high optical powers, and also marks the fact that in the low optical power limit where linear optomechanics is expected to work well, effects of coherent phonons do not appear. This also explains why this quantity has not been so far noticed in the context of quantum optomechanics. Because it does not show up anywhere in the corresponding fully linearized Langevin equations.

## Discussion

As shown in §S10 of supplementary information, a fairly convenient but approximate solution to the symmetrized spectral density of output optical field due to multiplicative noise is given as

13 $$\begin{array}{rcl}S(\omega ) & = & |{Y}_{11}(\omega {)|}^{2}{S}_{AA}(\omega )+\frac{1}{{\gamma }^{2}}{|[{Y}_{12}(\omega )+{Y}_{13}(\omega )]\ast \bar{a}(\omega )|}^{2}{S}_{BB}(\omega )\\  &  & +\,\frac{1}{{\theta }^{2}}{|[{Y}_{14}(\omega )\ast \overline{ab}(\omega )+{Y}_{15}(\omega )\ast \overline{a{b}^{\ast }}(\omega )]|}^{2}{S}_{BB}(\omega ),\end{array}$$

where spectral power densities *S*_*AA*_ and *S*_*BB*_ are already introduced in (S11) of supplementary information and convolutions * take place over the entire frequency axis. In practice it is far easier to use numerical integration, however, this can cause numerical instabilities when $|\omega -{\rm{\Delta }}|\, > \,\frac{1}{2}{\rm{\Omega }}$. Owing to the fractional polynomial expressions for the elements of scattering matrix elements as well as the multiplicative terms, it is possible to evaluate the integrals exactly using complex residue techniques. Here, we proceed using numerical integration of the convolution integrals. The third term involving the functions $\overline{ab}(\omega )$ and $\overline{a{b}^{\ast }}(\omega )$ are unnecessary for the 3 × 3 second-order formalism, and arise only in the 5 × 5 third-order formalism.

In the above equation, every term adds up the contribution from linear, second-order, and third-order optomechanics. These respectively are due to the processes of photon creation-annihilation $\{\hat{a},{\hat{a}}^{\dagger }\}$, the 1-photon/1-phonon blue $\{\hat{a}\hat{b},{\hat{a}}^{\dagger }{\hat{b}}^{\dagger }\}$ and red $\{\hat{a}{\hat{b}}^{\dagger },{\hat{a}}^{\dagger }\hat{b}\}$ processes, and the 1-photon/2-phonon second-order blue-like $\{\hat{a}{\hat{b}}^{2},{\hat{a}}^{\dagger }{\hat{b}}^{\dagger 2}\}$ and red-like $\{\hat{a}{\hat{b}}^{\dagger 2},{\hat{a}}^{\dagger }{\hat{b}}^{2}\}$ sideband processes. Apparently the Hermitian conjugate operators do not exist in the original 5 × 5 higher-order formalism (S70) of supplementary information, since they are completely uncoupled from their Hermitian counterparts. However, calculation of the noise spectral densities necessitates their presence, so that a real-valued and positive definite spectral density has actually already taken care of these conjugate processes. Obviously, the first term contributes to the *w* = Δ resonance, while the second term contributes to the first-order mechanical side-bands at *w* = Δ = ±Ω. Similarly, the third term constitutes the second-order mechanical sidebands at *w* = Δ = ±2Ω.

It has to be mentioned that the spectral density (13) is not mathematically exact, since the multiplicative operators appearing behind Weiner noise terms, are approximated by their time-averaged frequency-dependent terms (S68) of supplementary information.

**Figure 2:**
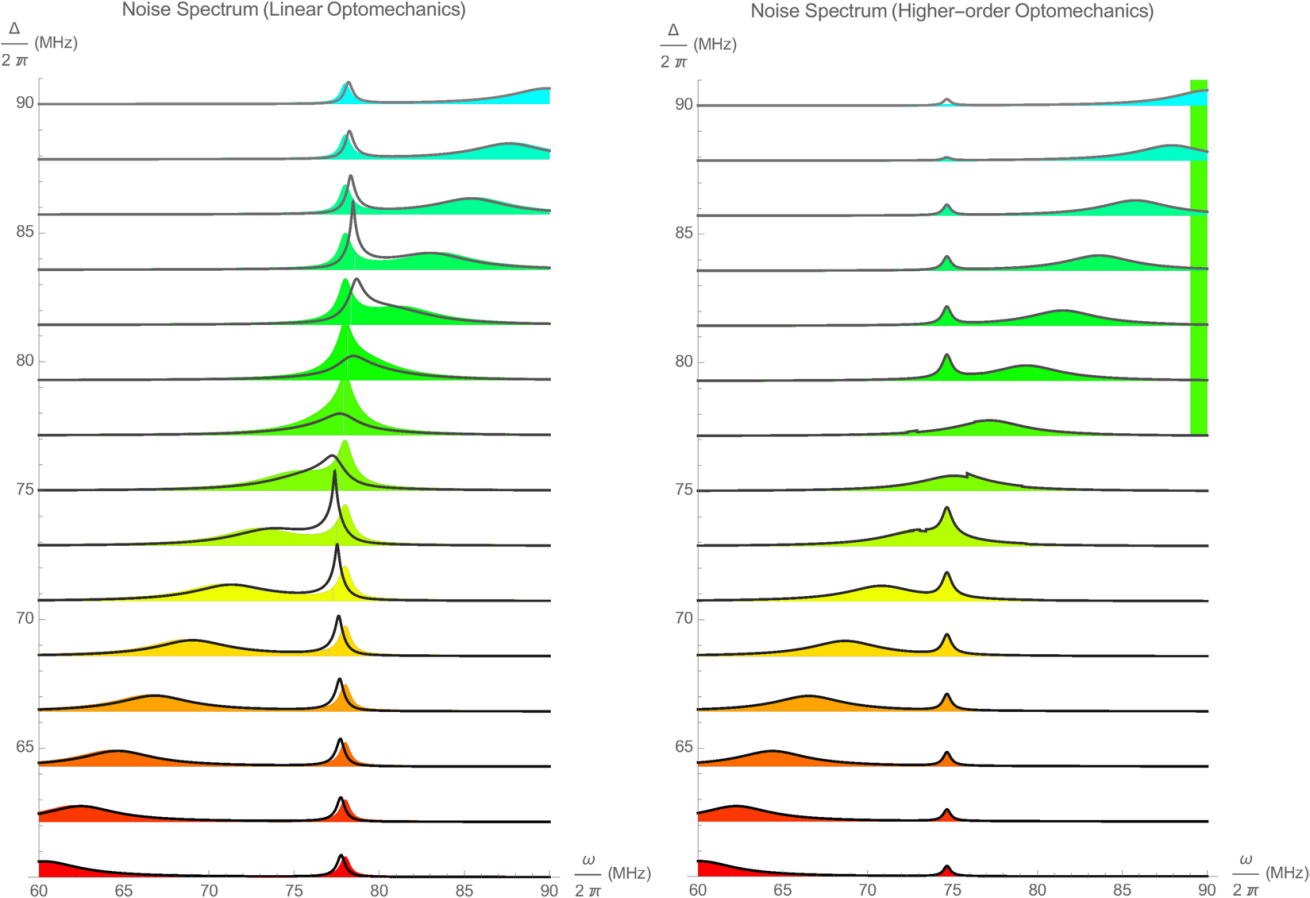
Noise spectrum across the red mechanical sideband and optical resonance for various detuning values ranging from Δ = 2*π* × 60 MHz to Δ = 2*π* × 90 MHz. Left panel corresponds to the simulations using 3 × 3 (color fills) and 4 × 4 linearized optomechanics (black lines). Right panel corresponds to the simulations using 3 × 3 (color fills) and 5 × 5 higher-order optomechanics (black lines). Optomechanical system parameters were taken from a remarkable experimental article^42^.

As an application example, we simulate the noise spectrum across the red mechanical sideband and optical resonance generated in an optomechanical experiment on the whispering galley mode of an optical micro-toroid, reported in a very remarkable experiment^42^. The pump is set around the red mechanical side band for various detuning values ranging from Δ = 2*π* × 60 MHz to Δ = 2*π* × 90MHz, and noise spectra are observed. In Fig. 2, the simulation results using linearized and higher-order optomechanics are illustrated. Here, the left panel shows the simulations using 3 × 3 linear optomechanics (color fills) with the basis $\{\hat{a},\hat{b},{\hat{b}}^{\dagger }\}$ and 4 × 4 linearized optomechanics (black lines) using the basis $\{\hat{a},{\hat{a}}^{\dagger },\hat{b},{\hat{b}}^{\dagger }\}$. While the linear 4 × 4 formalism is expected to be more accurate than the linear 3 × 3 formalism, there exists a notable difference between the two approaches. Here, the optomechanical parameters were taken from the same article with some adjustment to resemble the actual experiment^42^ as *T* = 65 mK, *P*_op_ = 1.4 mW, Ω = 2*π* × 78 MHz, *g*_0_ = 2*π* × 3.4 kHz, Γ = 2*π* × 407 kHz, *κ* = 2*π* × 3.54 MHz *η* = 0.5, and *λ* = 775 nm. On the right panel of Fig. 2 the simulations using 3 × 3 higher-order optomechanics (color fills) with the basis $\{\hat{a},\hat{a}\hat{b},\hat{a}{\hat{b}}^{\dagger }\}$ and 5 × 5 higher-order optomechanics (black lines) with the basis $\{\hat{a},\hat{a}\hat{b},\hat{a}{\hat{b}}^{\dagger },\hat{a}{\hat{b}}^{2},\hat{a}{\hat{b}}^{\dagger 2}\}$ are shown. Agreement between the second-order 3 × 3 and third-order 5 × 5 formalisms is remarkably good.

It is possible to employ the method of higher-order operators to investigate the dynamic stability of optomechanical systems in the side-band resolved operation limit. A stable optomechanical system can be still perturbed by thermal effects and they appear to be dominant in driving the cavity into instability for Doppler samples. However, for side-band resolved samples, thermal effects are much less pronounced and the major contribution to the instability comes from inherent nonlinear dynamics of the optomechanical interactions. That implies that optomechanical interactions are linearly stable, but they can become nonlinearly unstable at a certain interaction order to be discussed below.

The dynamic stability can be done by inspecting eigenvalues of the coefficients matrix [**M**]. If the real part of at least one of the eigenvalues is positive, then the system is unstable and its response to any perturbation grows indefinitely in time. The linear formalisms of optomechanics fails to describe this phenomenon, since they always yield constant eigenvalues. Even the second-order higher-operator method with 3 × 3 formalism, which describes the nonlinear 1-photon/1-phonon processes, fails to reproduce the correct expected stability phases. This only can be understood by employing at least the third-order 5 × 5 operator method, which includes the nonlinear 1-photon/2-phonon processes. Hence, surprisingly enough, it is the 1-photon/2-phonon process and beyond, which contributes to the unstability of an optomechanical system.

**Figure 3:**
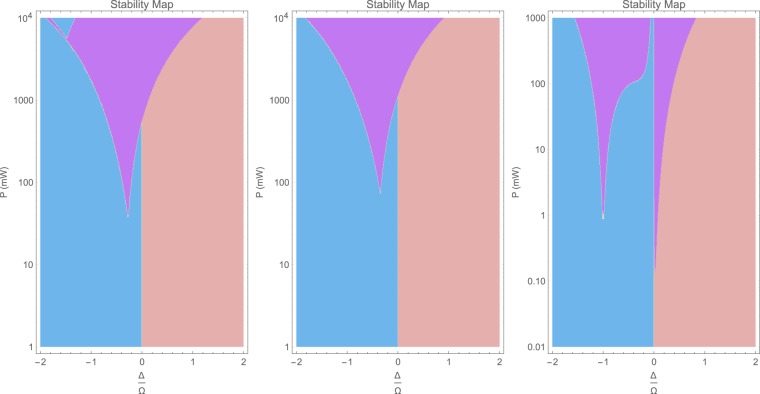
Dynamic stability phase map of a side-band resolved optomechanical systems studied in §S10 of supplementary information. Red and blue domains correspond to stable red ${\rm{\Delta }}\, > \,0$ and blue Δ < 0 detunings. The violet phase is the unstable region: Full numerical simulation of nonlinear dynamics (left); Boundary marked by (14) (middle); Linear stability map for the same system (Right).

To illustrate this, we calculate the stability phases of the side-band resolved system investigated in §S10 of supplementary information. Illustrated in Fig. 3, the stable and unstable regions of this systems across blue Δ < 0 and red ${\rm{\Delta }} > 0$ detunings versus input optical power *P*_op_ are illustrated. The v-shaped region in violet color, maps the unstable phase, while the red and blue colors correspond to the stable operation phases.

We also have calculated the linear stability from 4 × 4 full linear formalism, which appears on the right of Fig. 3. Not surprisingly, the linear and nonlinear stability diagrams remarkably are different. The linear instability starts at moderate resonant pump powers, while it starts rapidly growing exactly over the blue detuning at a slightly higher power.

Firstly, it can be seen that across almost the entire domains of linear stability, the system is also nonlinearly stable. Secondly, by observation of the nonlinear stability in the left and linear stability on the right, it can be seen that in most of the domain of linear stability, the system is already nonlinearly stable. Hence, any attempt to drive the system within the region of linearly unstable but nonlinearly stable, ultimately results in significant growth of mechanical amplitude and therefore side-bands. Any further increase in the amplitude of side-bands become limited due to nonlinear stability. Hence, four possible stability scenarios could be expected:

- Linearly and Nonlinearly Stable: The intersect of the linear and nonlinear stability domains, marks a shared domain of unconditionally stable optomechanical interaction. Unless the system is influenced by thermal or other nonideal effects, the stability is always guaranteed.
- Linearly Unstable, but Nonlinearly Stable: By inspection, an optomechanical system can be linearly unstable while nonlinearly stable. This corresponds to the domains where any attempt to drive the system in these regions causes immediate but limited growth in the amplitude of mechanical oscillations.
- Linearly and Nonlinearly Unstable: Under this scenario, the optomechanical cavity is always unstable regardless of the other nonideal effects. This happens only at remarkably high drive powers.
- Linear Stable, but Nonlinearly Unstable: The unlikely and surprising case of linear stability and nonlinear unstability is also possible according to the stability maps at some portions of non-resonant high drive powers. This strange behavior corresponds to the case when the system remains stable only at infinitesimal optical powers. Any fluctuation beyond tiny amplitudes shall drive the system into unstable growing and large amplitudes.

There is a threshold power *P*_th_ at which instabilites start to appear. For the side-band resolved case, this happens at *P*_th_ = 5 mW on the blue side. As it expected and in agreement to experimental observations, the unstable domain mostly covers the blue domain with Δ < 0. However, at higher optical powers than *P*_th_, instability phase can diffuse well into the red detunings as well ${\rm{\Delta }}\, > \,0$. For the cavity under consideration, this happens at much higher optical power of *P* = 14 *P*_th_ = 70 mW. Therefore, the general impression that instability always occurs on the blue side at every detuning above a certain threshold power is not correct. Interestingly, the boundary separating the dynamically stable and dynamically unstable phases for this side-band resolved sample with Ω ≫ *κ* ≫ Γ can be well estimated using

14 $$\bar{n}({\rm{\Delta }}) > {n}_{{\rm{cr}}},$$

in which *n*_cr_ is a critical cavity photon number. Extensive numerical tests for cavities within deep side-band resolved Ω ≫ *κ*, deep Doppler Ω ≫ *κ*, and intermediate ${\rm{\Omega }}\sim \kappa$ regimes reveal the existence of such a critical intracavity photon number limit *n*_cr_, beyond which dynamical instability takes over. However, for a cavity in deep side-band resolved regime, it can be estimated in a phenomenological way, and is roughly given by

15 $${n}_{{\rm{cr}}}\sim \frac{4}{{{\mathscr{C}}}_{0}}{(\frac{{\rm{\Omega }}}{\kappa })}^{2}\mathrm{.}$$

Here, ${{\mathscr{C}}}_{0}$ is the single-photon cooperativity given by

16 $${{\mathscr{C}}}_{0}={g}_{0}^{2}/\kappa {\rm{\Gamma }}\mathrm{.}$$

If the cavity is not side-band resolved, (15) cannot be used, but numerical computations can still yield the limiting number *n*_cr_.

For Doppler cavities, no such dynamic instability can be observed, and therefore thermal effects should dominate over dynamical effects in driving a Doppler cavity toward instability. Meanwhile, it is the nonlinear optomechanical dynamics which seems to be dominant in driving a side-band resolved cavity into instability. As a result, the existence of such a critical maximum intracavity photon number is not related to thermal effects, but rather to the nonlinear stability.

## Methods

For an extensive description of theoretical methods, refer to the Supplementary Information.

## Conclusions

A new analytical method was shown to solve the standard optomechanical interaction with cubic nonlinearity interaction, based on the higher-order operators. It was demonstrated that not only the higher-order operator method can reproduce the linear optomechanics, but also it can predict and provide estimates to unnoticed effects such as a new type of symmetry breaking in frequency, here referred to as side-band inequivalence, and yield new explicit expressions for quantities such as the coherent phonon population and higher-order spring effect. Corrections to the standard spring effect due to higher-order interactions have been found, and it has been shown that such corrections arise mainly because of the coherent phonons and can significantly influence measurement of single-photon optomechanical interaction rate through spring effect. A minimal basis has been defined which allows exact and explicit solution to standard nonlinear optomechanics, using the method of higher-order operators. This method can be finally used to investigate the dynamic nonlinear stability of optomechanical systems, and it has been demonstrated that at least the third-order nonlinear processes are prerequisite for occurrence of dynamic instability. We have shown that there is a reasonable correspondence between the onset of nonlinear dynamic instability and a critical intracavity photon number limit, which remains independent of thermal effects.

## Electronic supplementary material

Supplementary Information

Mathematical Codes
