# Supplementary material for: Method of Higher-order Operators for Quantum Optomechanics
Source: Sci Rep. 2018 Aug 1;8:11566. doi: 10.1038/s41598-018-30068-7 (PMC6070579; doi:10.1038/s41598-018-30068-7)
Supplement: Supplementary file 1 — Supplementary Information [file 41598_2018_30068_MOESM1_ESM.pdf]

Supplementary Information: Theoretical Methods

Method of Higher-order Operators for Quantum Optomechanics

Sina Khorasani

S1 Optomechanical Hamiltonian

The Langevin equations for the Hamiltonian $\mathbb{H}_{\text{OM}} = \hbar\Omega\hat{m} - \hbar\Delta\hat{n} - \hbar g_0\hat{n}(\hat{b}+\hat{b}^\dagger)$ with the basis $\{A\}^{\text{T}} = \left\{\hat{a},\hat{b},\hat{a}\hat{b},\hat{a}\hat{b}^\dagger,\hat{n},\hat{c}\right\}$ are given exactly by

$$\begin{bmatrix} i\Delta-\frac{\kappa}{2} & 0 & ig_0 & ig_0 & 0 & 0 \\ 0 & -(i\Omega+\frac{\Gamma}{2}) & 0 & 0 & ig_0 & 0 \\ ig_0\,(\hat{m}+\hat{n}+1) & 0 & -i\left(\Omega-\Delta-g_0\hat{b}\right)-\frac{\gamma}{2} & 0 & 0 & 0 \\ ig_0\,(\hat{m}-\hat{n}) & 0 & 0 & i\left(\Omega+\Delta+g_0\hat{b}^\dagger\right)-\frac{\gamma}{2} & 0 & 0 \\ 0 & 0 & 0 & 0 & -\kappa & 0 \\ 0 & 0 & ig_0\hat{a} & ig_0\hat{a} & 0 & 2i[\Delta+g_0(\hat{b}+\hat{b}^\dagger)]-\kappa \end{bmatrix}$$
$$\times\left\{\begin{array}{c} \hat{a} \\ \hat{b} \\ \hat{a}\hat{b} \\ \hat{a}\hat{b}^\dagger \\ \hat{n} \\ \hat{c} \end{array}\right\} - \left\{\begin{array}{c} \sqrt{\kappa}\hat{a}_{\text{in}} \\ \sqrt{\Gamma}\hat{b}_{\text{in}} \\ \sqrt{\gamma}\big(\hat{a}\hat{b}\big)_{\text{in}} \\ \sqrt{\gamma}\big(\hat{a}\hat{b}^\dagger\big)_{\text{in}} \\ \sqrt{2\kappa}\hat{n}_{\text{in}} \\ \sqrt{2\kappa}\hat{c}_{\text{in}} \end{array}\right\} = \frac{d}{dt}\left\{\begin{array}{c} \hat{a} \\ \hat{b} \\ \hat{a}\hat{b} \\ \hat{a}\hat{b}^\dagger \\ \hat{n} \\ \hat{c} \end{array}\right\}, \quad \text{(S1)}$$

in which $\gamma = \kappa+\Gamma$. We have set $\hat{x}=\hat{a}$ in all equations except the second and third where both of the bath operators $\hat{x}=\hat{a}$ and $\hat{x}=\hat{b}$ are taken separately to construct the noise terms, $\sqrt{2}\hat{n}_{\text{in}} = \hat{a}^\dagger\hat{a}_{\text{in}} + \hat{a}^\dagger_{\text{in}}\hat{a}$, $(\hat{a}\hat{b}^\dagger)_{\text{in}} = \hat{a}_{\text{in}}\hat{b}^\dagger + \hat{a}\hat{b}^\dagger_{\text{in}}$, $(\hat{a}\hat{b})_{\text{in}} = \hat{a}_{\text{in}}\hat{b} + \hat{a}\hat{b}_{\text{in}}$ and $\sqrt{2}\hat{c}_{\text{in}} = \hat{a}\hat{a}_{\text{in}}$.

The system (S1) is still nonlinear and non-integrable because of the dependence of the coefficients matrix on the operators. But it can be simplified by first noting that from the fifth equation we could expect any disturbance in $\hat{n}$ would decay as $\delta\hat{n}(t)\sim\exp(-\kappa t)$ on time scales smaller than κ^{-1}. This can be further approximated as $\hat{n}\sim\bar{n}$ at steady input. Similar argument goes with $\delta\hat{m}\sim\exp(-\Gamma t)$ in response to a disturbance on time scales smaller than Γ^{-1}, which enables us to make the approximate replacement $\hat{m}\sim\bar{m}$ at equilibrium.

For the phononic mechanical operators $\hat{b}$ and $\hat{b}^\dagger$ appearing within the brackets, approximate decays $\delta\hat{b}(t)\sim\exp[-(i\Omega+\frac{1}{2}\Gamma)t]$ and $\delta\hat{b}^\dagger(t)\sim\exp[(i\Omega-\frac{1}{2}\Gamma)t]$ in response to disturbances hold, making the coefficients matrix time-dependent. But these can be nevertheless dropped in whole if we notice that $g_0\bar{b} << \Omega$ which is the normal experimental condition of weakly-coupling in optomechanics. Otherwise, they can replaced by constant amplitudes $\bar{b}$ and $\bar{b}^*$ given below in (S6) on sufficiently longer time scales than Γ^{-1} for strongly-coupled systems.

Such types of approximations are in fact quite highly in use within the context of continuous wave standard optomechanics. Therefore, once the steady state solution to (S1) around the equilibrium values due to optical drive $\langle\hat{a}_{\text{in}}\rangle=\alpha$ is sought, the coefficients matrix can be kept time-independent, keeping only the fluctuations of input terms as the only source. The case of time dependent drive $\alpha=\alpha(t)$ for pulsed experiments shall be discussed later in the article.

Having said that, all the operators $\hat{n}$, $\hat{m}$, $\hat{b}$, and $\hat{b}^\dagger$ in the coefficients matrix can be replaced by their respective average values to proceed with the second-order accurate optomechanical system of equations as

$$\begin{bmatrix} i\Delta-\frac{\kappa}{2} & 0 & ig_0 & ig_0 & 0 & 0 \\ 0 & -(i\Omega+\frac{\Gamma}{2}) & 0 & 0 & ig_0 & 0 \\ iL^+ & 0 & -i\left(\Omega-\Delta-s\right)-\frac{\gamma}{2} & 0 & 0 & 0 \\ iL^- & 0 & 0 & i\left(\Omega+\Delta+s^*\right)-\frac{\gamma}{2} & 0 & 0 \\ 0 & 0 & 0 & 0 & -\kappa & 0 \\ 0 & 0 & ig & ig & 0 & 2i(\Delta+2\Re[s])-\kappa \end{bmatrix}$$
$$\times\left\{\begin{array}{c} \hat{a} \\ \hat{b} \\ \hat{a}\hat{b} \\ \hat{a}\hat{b}^\dagger \\ \hat{n} \\ \hat{c} \end{array}\right\} - \left\{\begin{array}{c} \sqrt{\kappa}\hat{a}_{\text{in}} \\ \sqrt{\Gamma}\hat{b}_{\text{in}} \\ \sqrt{\gamma}\big(\hat{a}\hat{b}\big)_{\text{in}} \\ \sqrt{\gamma}\big(\hat{a}\hat{b}^\dagger\big)_{\text{in}} \\ \sqrt{2\kappa}\hat{n}_{\text{in}} \\ \sqrt{2\kappa}\hat{c}_{\text{in}} \end{array}\right\} = \frac{d}{dt}\left\{\begin{array}{c} \hat{a} \\ \hat{b} \\ \hat{a}\hat{b} \\ \hat{a}\hat{b}^\dagger \\ \hat{n} \\ \hat{c} \end{array}\right\}, \quad \text{(S2)}$$

in which $g = g_0\sqrt{\bar{n}}$, $s = g_0\bar{b}$ with $\Re[\bar{b}] = \bar{x}/2x_{\rm zp}$ and $x_{\rm zp}$ being the zero-point displacement, $L^+ = g_0(\bar{m}+\bar{n}+1)$, and $L^- = g_0(\bar{m}-\bar{n})$. These can be further approximated by $L^\pm \approx \pm g_0\bar{n} = \pm F$ under normal experimental conditions of an ultracold cavity with sufficiently high pumping. The average mirror displacement $\bar{x}$ is due to the average radiation pressure. The fact that $L^+ \neq -L^-$ provides the quantum mechanical asymmetry between blue and red sidebands.

It is easy to verify that this way of linearization decouples the state operators and reduces the space into a 3-dimensional one spanned by $\{A\}^{\rm T} = \{\hat{a}, \hat{a}\hat{b}, \hat{a}\hat{b}^\dagger\}$. This will be discussed in further details later.

In the absence of red-side-band optical cooling tone as well as any other interaction, the average population value is $\bar{m} = 1/[\exp(\hbar\Omega/k_{\rm B}T)-1]$, while $\bar{n}$ can be obtained from the steady state solution of the first row by replacements of input noise term $\sqrt{\kappa}\hat{a}_{\rm in} \to \alpha + \sqrt{\kappa}\hat{a}_{\rm in}$. Here, α is the input photon flux originally due to an undisplayed resonant drive term $\mathbb{H}_{\rm d} = \hbar(\alpha\hat{a}+\alpha^*\hat{a}^\dagger)$ added to the Hamiltonian $\mathbb{H}_{\rm OM}$. Furthermore, α has some non-zero phase taken from the cavity population $\bar{n}$ away. Now that the drive term $\mathbb{H}_{\rm d}$ has been dropped from $\mathbb{H}_{\rm OM}$, and $\hat{a}_{\rm in}$ now only contains the fluctuations with zero-average $\langle\hat{a}_{\rm in}\rangle = 0$.

As it will be shown later, the quantity $\bar{m}$ here being referred to as the coherent phonon population, can enter the optomechanical interaction processes due to higher-order effects, where its value normally needs to be fitted for cavities in the Doppler regime. Hence, the coherent phonon population $\bar{m}$ is independent of the simple thermal equilibrium value m, and actually represents those number of phonons who take part in the optomechanical interaction. We will observe that the negative detunings with the blue process can actually lead to a relatively constant phonon population, whereas on the red detunings it starts to decrease with the detuning.

Defining $K = \bar{n}\kappa$ we may use the substitutions for the noise and input terms as

$$\begin{aligned}
\sqrt{\gamma}\left(\hat{a}\hat{b}\right)_{\rm in} &\to \sqrt{\Gamma\bar{n}}\hat{b}_{\rm in} + \sqrt{\kappa}\bar{b}\hat{a}_{\rm in} + \bar{b}\alpha, \\
\sqrt{\gamma}\left(\hat{a}\hat{b}^\dagger\right)_{\rm in} &\to \sqrt{\Gamma\bar{n}}\hat{b}_{\rm in}^\dagger + \sqrt{\kappa}\bar{b}^*\hat{a}_{\rm in} + \bar{b}^*\alpha, \\
\sqrt{\kappa}\hat{n}_{\rm in} &\to \sqrt{K}\hat{a}_{\rm in} + \sqrt{K}\hat{a}_{\rm in}^\dagger + 2\sqrt{\bar{n}}\Re[\alpha], \\
\sqrt{\kappa}\hat{c}_{\rm in} &\to \sqrt{K}\hat{a}_{\rm in} + \sqrt{\bar{n}}\alpha.
\end{aligned} \tag{S3}$$

These substitutions follow the fact that terms such as $\hat{a}\hat{a}_{\rm in}$ which contain the interaction of a time-dependent operator $\hat{a}(t)$ and a purely white Weiner noise process with zero average $\langle\hat{a}_{\rm in}\rangle = 0$, can be fairly well approximated by noting first that $\hat{a}(t) \sim \bar{a}\exp(i\Delta t)$ around the equilibrium, and then noting that shifting the noise process $\hat{a}_{\rm in}$ in frequency to the amount of Δ has essentially no effect by definition. Hence, the sinusoidal time dependence $\exp(i\Delta t)$ is irrelevant and can be dropped. Similar arguments hold for the phononic operator $\hat{b}(t) \sim \bar{b}\exp(-i\Omega t)$ and their Hermitian adjoints interacting with a white noise term with uniform spectrum.

This allows us to ultimately rewrite the Langevin equations (S2) as

$$\begin{bmatrix}
i\Delta-\frac{\kappa}{2} & 0 & ig_0 & ig_0 & 0 & 0 \\
0 & -(i\Omega+\frac{\Gamma}{2}) & 0 & 0 & ig_0 & 0 \\
iL^+ & 0 & -i(\Omega-\Delta-s)-\frac{\gamma}{2} & 0 & 0 & 0 \\
iL^- & 0 & 0 & i(\Omega+\Delta+s^*)-\frac{\gamma}{2} & 0 & 0 \\
0 & 0 & 0 & 0 & -\kappa & 0 \\
0 & 0 & ig & ig & 0 & 2i(\Delta+2\Re[s])-\kappa
\end{bmatrix}$$

$$\times\left\{\begin{matrix}\hat{a}\\ \hat{b}\\ \hat{a}\hat{b}\\ \hat{a}\hat{b}^\dagger\\ \hat{n}\\ \hat{c}\end{matrix}\right\} - \begin{bmatrix}
\sqrt{\kappa} & 0 & 0 & 0 \\
0 & 0 & \sqrt{\Gamma} & 0 \\
\sqrt{\kappa}\bar{b} & 0 & \sqrt{\Gamma\bar{n}} & 0 \\
\sqrt{\kappa}\bar{b}^* & 0 & 0 & \sqrt{\Gamma\bar{n}} \\
\sqrt{K} & \sqrt{K} & 0 & 0 \\
\sqrt{K} & 0 & 0 & 0
\end{bmatrix}\left\{\begin{matrix}\hat{a}_{\rm in}\\ \hat{a}_{\rm in}^\dagger\\ \hat{b}_{\rm in}\\ \hat{b}_{\rm in}^\dagger\end{matrix}\right\} - \begin{bmatrix}
1 & 0 \\
0 & 0 \\
\bar{b} & 0 \\
\bar{b}^* & 0 \\
\sqrt{\bar{n}} & \sqrt{\bar{n}} \\
\sqrt{\bar{n}} & 0
\end{bmatrix}\left\{\begin{matrix}\alpha\\ \alpha^*\end{matrix}\right\} = \frac{d}{dt}\left\{\begin{matrix}\hat{a}\\ \hat{b}\\ \hat{a}\hat{b}\\ \hat{a}\hat{b}^\dagger\\ \hat{n}\\ \hat{c}\end{matrix}\right\}. \tag{S4}$$

The second term on the right is the noise fluctuations due to the optical and mechanical fields with zero average $\langle\hat{a}_{\rm in}\rangle = \langle\hat{b}_{\rm in}\rangle = 0$, and the last term in the above is proportional to the input photon flux $|\alpha| = \sqrt{\varepsilon\kappa P/\hbar\omega}$ where P is the incident radiation power and ε is the coupling efficiency. As it will be mentioned briefly later, the average values $\bar{n}$ and $\bar{x}$ have to be solved by setting $d/dt = 0$ on the left and taking average values, which eliminates the noise fluctuations, causing the replacements $\hat{a} \to \sqrt{\bar{n}}$, $\hat{b} \to \bar{b}$, $\hat{a}\hat{b} \to \bar{b}\sqrt{\bar{n}}$, $\hat{a}\hat{b}^\dagger \to \bar{b}^*\sqrt{\bar{n}}$, $\hat{n} \to \bar{n}$, and $\hat{c} \to \bar{n}/2$.

Hence, the average values $\bar{a} = \sqrt{\bar{n}}$ and $\bar{b}$ get nonlinearly coupled to the input flux α through the system of algebraic relations

as

$$\begin{bmatrix} i\Delta-\frac{\kappa}{2} & 0 & ig_0 & ig_0 & 0 & 0 \\ 0 & -(i\Omega+\frac{\Gamma}{2}) & 0 & 0 & ig_0 & 0 \\ iL^+ & 0 & -i\left(\Omega-\Delta-g_0\bar{b}\right)-\frac{\gamma}{2} & 0 & 0 & 0 \\ iL^- & 0 & 0 & i\left(\Omega+\Delta+g_0\bar{b}^*\right)-\frac{\gamma}{2} & 0 & 0 \\ 0 & 0 & 0 & 0 & -\kappa & 0 \\ 0 & 0 & ig & ig & 0 & i[\Delta+g_0(\bar{b}+\bar{b}^*)]-\frac{\kappa}{2} \end{bmatrix}$$

$$\times\left\{\begin{array}{c} \bar{a} \\ \bar{b} \\ \bar{a}\bar{b} \\ \bar{a}\bar{b}^* \\ \bar{a}^2 \\ \bar{a}^2 \end{array}\right\}=\left\{\begin{array}{cc} 1 & 0 \\ 0 & 0 \\ \bar{b} & 0 \\ \bar{b}^* & 0 \\ \bar{a} & \bar{a} \\ \bar{a} & 0 \end{array}\right\}\left\{\begin{array}{c} \alpha \\ \alpha^* \end{array}\right\}. \tag{S5}$$

With a given input photon flux $|\alpha|$, this system can be now solved to obtain the phase $\angle\alpha$ in such a way that $\angle\bar{a}=0$. Then $\bar{a}$ and $\bar{b}$ can be obtained in an algebraic manner. This sets up a system of equations in terms of the total of four unknowns $\angle\alpha$, $\bar{a}=\sqrt{\bar{n}}$, $\bar{b}$, and $\bar{b}^*$.

In the above system, the second equation is independent of α, while together the fifth they yield

$$\begin{aligned} \bar{b} &= \frac{ig_0}{i\Omega+\frac{1}{2}\Gamma}\bar{a}^2, \\ \bar{a} &= -\frac{1}{\kappa}(\alpha+\alpha^*). \end{aligned} \tag{S6}$$

This also already solves $\bar{b}^*$ in terms of $\bar{a}$. Plugging in the results into the first equation leads to the third-order algebraic equation which can be now solved. Doing this and some algebraic manipulation gives the equation

$$ig_0^2\frac{2\Omega}{\Omega^2+\frac{1}{4}\Gamma^2}\bar{a}^3+\left(i\Delta-\frac{\kappa}{2}\right)\bar{a}=\alpha. \tag{S7}$$

This equation in general is expected to yield only real-valued $\bar{a}$. Separating the real and imaginary parts gives

$$\begin{aligned} \Re[\alpha] &= -\kappa\frac{\bar{a}}{2} \\ \Im[\alpha] &= g_0^2\frac{2\Omega}{\Omega^2+\frac{1}{4}\Gamma^2}\bar{a}^3+\Delta\bar{a}. \end{aligned} \tag{S8}$$

The first of these is the same as the second of (S6). The above two equations can be now iteratively solved to yield $\angle\alpha$ and $\bar{a}$ for a given $|\alpha|$. One may also discard $\angle\alpha$ by combining the above two, resulting in

$$|\alpha|^2=\left[\frac{\kappa^2}{4}+\left(\frac{2g_0^2\Omega}{\Omega^2+\frac{1}{4}\Gamma^2}\bar{n}+\Delta\right)^2\right]\bar{n}. \tag{S9}$$

Only real and positive-valued roots of (S9) for $\bar{n}$ are acceptable. Sufficiently large blue-detuning with $\Delta<\Delta_{\rm b}<0$ causes the well-known bistability. It is easy to find the negative blue detuning $\Delta_{\rm b}<0$ at which bistability starts to appear, by looking for the only negative real root of the cubic equation

$$-\Delta_{\rm b}\left(\Delta_{\rm b}^2+\frac{9}{4}\kappa^2\right)=\frac{27g_0^2\Omega}{\Omega^2+\frac{1}{4}\Gamma^2}|\alpha|^2. \tag{S10}$$

These two noise terms we assume have the flat shot-noise uncorrelated spectral power densities

$$\begin{aligned} S_{AA}(\omega) &= \frac{1}{2}, \\ S_{BB}(\omega) &= m+\frac{1}{2}, \end{aligned} \tag{S11}$$

which are identical on both positive and negative frequencies. The ultimate difference of noise power spectral densities will be later maintained by the asymmetry caused by $L^+-L^-=2F+g_0>0$. Here, $m=1/\left[\exp(\hbar\Omega/k_{\rm B}T)-1\right]$ is the population of incoherent phonons under thermal equilibrium, which contribute to the random fluctuations of thermal noise. This quantity is not to be mistaken with $\bar{m}$ which here denotes the population of coherent phonons, contributing coherently to the optomechanical interaction, and are driven by the optical radiation pressure. This shall be discussed later in §S6.

S1.1 Perturbative Solution

At this moment, the system of equations (S4) can be perturbed around equilibrium values found above. This procedure and taking a Fourier transform gives out the solution. Let us define first

$$\mathbf{M}=\left[\begin{array}{cccccc} i\Delta-\frac{\kappa}{2} & 0 & ig_0 & ig_0 & 0 & 0 \\ 0 & -(i\Omega+\frac{\Gamma}{2}) & 0 & 0 & ig_0 & 0 \\ iL^{+} & 0 & -i\left(\Omega-\Delta-s\right)-\frac{\gamma}{2} & 0 & 0 & 0 \\ iL^{-} & 0 & 0 & i\left(\Omega+\Delta+s^{*}\right)-\frac{\gamma}{2} & 0 & 0 \\ 0 & 0 & 0 & 0 & -\kappa & 0 \\ 0 & 0 & ig & ig & 0 & 2i(\Delta+2\Re[s])-\kappa \end{array}\right], \tag{S12}$$

as well as

$$\begin{aligned} \left\{\delta A(\omega)\right\}^{\mathrm{T}} &= \left\{\delta\hat{a}(\omega),\delta\hat{b}(\omega),\delta(\hat{a}\hat{b})(\omega),\delta(\hat{a}\hat{b}^{\dagger})(\omega),\delta\hat{n}(\omega),\delta\hat{c}(\omega)\right\}, \\ \left\{A_{\mathrm{in}}(\omega)\right\}^{\mathrm{T}} &= \left\{\hat{a}_{\mathrm{in}}(\omega),\hat{a}_{\mathrm{in}}^{\dagger}(\omega),\hat{b}_{\mathrm{in}}(\omega),\hat{b}_{\mathrm{in}}^{\dagger}(\omega)\right\}, \\ \left[\sqrt{\Gamma}\right] &= \left[\begin{array}{cccc} \sqrt{\kappa} & 0 & 0 & 0 \\ 0 & 0 & \sqrt{\Gamma} & 0 \\ \sqrt{\kappa}\bar{b} & 0 & \sqrt{\Gamma\bar{n}} & 0 \\ \sqrt{\kappa}\bar{b}^{*} & 0 & 0 & \sqrt{\Gamma\bar{n}} \\ \sqrt{K} & \sqrt{K} & 0 & 0 \\ \sqrt{K} & 0 & 0 & 0 \end{array}\right]. \end{aligned} \tag{S13}$$

Then, taking $\mathbf{I}_j$ as the $j\times j$ identity matrix, we get

$$\begin{aligned} \left\{A_{\mathrm{out}}(\omega)\right\} &= \left\{A_{\mathrm{in}}(\omega)\right\}-\left[\sqrt{\Gamma}\right]^{\mathrm{T}}\left\{\delta A(\omega)\right\}=\mathbf{Y}(\omega)\left\{A_{\mathrm{in}}(\omega)\right\}, \\ \left\{\delta A(\omega)\right\} &= \mathbf{Z}(\omega)\left\{A_{\mathrm{in}}(\omega)\right\}, \\ \mathbf{Z}(\omega) &= \left[\mathbf{M}-i\omega\mathbf{I}_6\right]^{-1}\left[\sqrt{\Gamma}\right], \\ \mathbf{Y}(\omega) &= \mathbf{I}_4-\left[\sqrt{\Gamma}\right]^{\mathrm{T}}\mathbf{Z}(\omega). \end{aligned} \tag{S14}$$

Here, $\mathbf{Y}(\omega)$ is the scattering matrix connecting the input and output ports. Now, the spectral density of reflected light from the cavity can be found using (S11) by the expression

$$S(\omega)=\left[|Y_{11}(\omega)|^2+|Y_{12}(\omega)|^2\right]S_{AA}(\omega)+\left[|Y_{13}(\omega)|^2+|Y_{14}(\omega)|^2\right]S_{BB}(\omega), \tag{S15}$$

as long as the noise processes of $\hat{a}_{\mathrm{in}}$ and $\hat{b}_{\mathrm{in}}$ have zero cross-correlation [S1].

S2 Linearized Optomechanics

It is fairly easy to see that the system of equations (S19) when simplified and rewritten for the basis $\{\hat{a},\hat{b},\hat{b}^{\dagger}\}$ reproduces the widely used linearized optomechanical equations [S2]. To demonstrate this, we ignore the perturbation matrix $\delta\mathbf{N}$, as well as $\bar{b}/\sqrt{\bar{n}}$ in the noise terms, and then employ the substitutions

$$\begin{aligned} \hat{a}\hat{b} &\rightarrow \exp\left[(i\Delta-\frac{1}{2}\kappa)t\right]\bar{a}\hat{b}=\exp\left[(i\Delta-\frac{1}{2}\kappa)t\right]\sqrt{\bar{n}}\hat{b}, \\ \hat{a}\hat{b}^{\dagger} &\rightarrow \exp\left[(i\Delta-\frac{1}{2}\kappa)t\right]\bar{a}\hat{b}^{\dagger}=\exp\left[(i\Delta-\frac{1}{2}\kappa)t\right]\sqrt{\bar{n}}\hat{b}^{\dagger}. \end{aligned} \tag{S16}$$

This will immediately result in rewriting (S19) as

$$\frac{d}{dt}\left\{\begin{array}{c}\delta\hat{a}\\ \delta\hat{b}\\ \delta\hat{b}^{\dagger}\end{array}\right\}=\left[\begin{array}{ccc} i\Delta-\frac{\kappa}{2} & ig_0 & ig_0 \\ 0 & -i\Omega-\frac{\Gamma}{2} & 0 \\ 0 & 0 & i\Omega-\frac{\Gamma}{2}\end{array}\right]\left\{\begin{array}{c}\delta\hat{a}\\ \delta\hat{b}\\ \delta\hat{b}^{\dagger}\end{array}\right\}+\left[\begin{array}{ccc}\sqrt{\kappa} & 0 & 0\\ 0 & \sqrt{\Gamma} & 0\\ 0 & 0 & \sqrt{\Gamma}\end{array}\right]\left\{\begin{array}{c}\hat{a}_{\mathrm{in}}\\ \hat{b}_{\mathrm{in}}\\ \hat{b}_{\mathrm{in}}^{\dagger}\end{array}\right\}, \tag{S17}$$

which is nothing but exactly the linearized state equations of optomechanics. Hence, the method of higher-order operators [S3] is mathematically able to reproduce the less approximate linearized optomechanics.

S3 Pulsed Drive

Under the situation of pulsed drive, one may assume the input photon rate $\alpha(t)$ to be a function of time. If the input drive varies on a time-scale or longer than the mechanical period with $|d\alpha(t)/dt| < \Omega\alpha$, then one may assume $\bar{n}(t)$ is solved through (S9) at each moment with updated momentary mechanical frequency $\Omega(t)$ and linewidth $\Gamma(t)$ to yield an effective time dependent coefficients matrix $\mathbf{M}(t)$. This offers the solution

$$\begin{aligned}
\{A(t)\} &= \exp\left[\int_0^t \mathbf{M}(\tau)d\tau\right]\{A(0)\} + \int_0^t \exp\left[\int_0^{t-\tau}\mathbf{M}(\nu)d\nu\right][\beta(\tau)]\{\alpha(\tau)\}d\tau \\
&+ \int_0^t \exp\left[\int_0^{t-\tau}\mathbf{M}(\nu)d\nu\right][\Gamma(\tau)]\{A_{\text{in}}(\tau)\}d\tau \\
[\beta(t)]^{\text{T}} &= \begin{bmatrix} 1 & 0 & \bar{b}(t) & \bar{b}^*(t) & \sqrt{\bar{n}(t)} & \sqrt{\bar{n}(t)} \\ 0 & 0 & 0 & \sqrt{\bar{n}(t)} & 0 & \end{bmatrix}, \\
\{\alpha(t)\}^{\text{T}} &= \{\alpha(t), \alpha^*(t)\}.
\end{aligned} \tag{S18}$$

S4 Side-band Inequivalence

Let us go back to the set of equations (S12) and only retain the first, third, and fourth equations. This reduction gives a 3×3 system of equations, identical to (S14) with the redefinitions

$$\begin{aligned}
\mathbf{M} &= \mathbf{N} + \delta\mathbf{N} \\
\mathbf{N} &= \begin{bmatrix} i\Delta - \frac{\kappa}{2} & ig_0 & ig_0 \\ iF & -i(\Omega-\Delta) - \frac{\gamma}{2} & 0 \\ -iF & 0 & i(\Omega+\Delta) - \frac{\gamma}{2} \end{bmatrix}, \\
\{\delta A(\omega)\}^{\text{T}} &= \left\{\delta\hat{a}(\omega), \delta(\hat{a}\hat{b})(\omega), \delta(\hat{a}\hat{b}^\dagger)(\omega)\right\}, \\
\{A_{\text{in}}(\omega)\}^{\text{T}} &= \left\{\hat{a}_{\text{in}}(\omega), \hat{b}_{\text{in}}(\omega), \hat{b}^\dagger_{\text{in}}(\omega)\right\}, \\
\left[\sqrt{\Gamma}\right] &= \begin{bmatrix} \sqrt{\kappa} & 0 & 0 \\ \sqrt{\kappa}\bar{b} & \sqrt{\Gamma\bar{n}} & 0 \\ \sqrt{\kappa}\bar{b}^* & 0 & \sqrt{\Gamma\bar{n}} \end{bmatrix}.
\end{aligned} \tag{S19}$$

Here, the perturbation matrix $\delta\mathbf{N}$ is defined through the relation

$$\delta\mathbf{N} = \begin{bmatrix} 0 & 0 & 0 \\ if^+ & is & 0 \\ if^- & 0 & is^* \end{bmatrix}, \tag{S20}$$

in which $f^+ = g_0(\bar{m}+1)$ and $f^- = g_0\bar{m}$. It is quite apparent that the second and third rows of $\mathbf{N}$ in (S19) are complex conjugates.

By setting $\Delta = 0$ in (S19) one would expect identically displaced sidebands at $\pm\Omega$. However, this is contingent on the fact that the eigenvalues of $\mathbf{N}$ be either complex conjugates as $\Im[\eta] = \pm\Omega$ corresponding to the frequencies of the sidebands, or $\Im[\eta] = 0$ corresponding to the resonant pump. However, the presence of perturbation matrix $\delta\mathbf{N}$ breaks this symmetry between the sidebands. This causes a very tiny displacement of sidebands so that $\Delta_{\text{r}} + \Delta_{\text{b}} \neq 0$. First figure of the main article illustrates the side-band asymmetry for various intracavity photon numbers $\bar{n} = (g/g_0)^2$ and coherent phonon numbers $\bar{m}$, when $g_0/\Omega = 10^{-3}$. This effect is actually due to the higher-order optomechanical spring effect analyzed in the following.

It has to be noticed that the horizontal axes are nonlinear functions of the incident light intensity and therefore α. Typically, an inequivalence would be observable in a heterodyne side-band resolved experiment if the effect is large enough to allow clear and measurable motion of side-bands. This condition requires $|\Delta_{\text{r}} + \Delta_{\text{b}}| > \Gamma = \Omega/Q_{\text{m}}$, in which Q_{m} is the mechanical quality factor. If $Q_{\text{m}} > 10^5$, then an intracavity occupation number of $\bar{n} > 10^4$ should be sufficient to detect any such inequivalence.

The side-band inequivalence should not be mistaken with the fundamental energy conservation and time reversal symmetry. Firstly according to these, the spectral density on the negative frequencies of the spectrum should be mirror symmetric with respect to the positive frequencies. Normally, the actual optical frequency ω is much larger than the mechanical frequency Ω, so that the observed red- and blue-detuned sidebands within the range $\Delta \in (-\Omega, +\Omega)$ actually entirely correspond to the positive absolute frequencies. So the speculation that $\delta\Delta$ could be non-zero has nothing to do with the time-reversal symmetry. Secondly, side-band inequivalence is a purely nonlinear effect and is therefore strictly forbidden in any linearized approximation of the optomechanical Hamiltonian.

S5 Resonance Shift

The contribution of the terms $\pm iF + if^{\pm}$ to the mechanical frequency Ω in the second and third equations of (S20), can be held responsible for the so-called optomechanical spring effect [S1, S2, S4–S10]. As the result of optomechanical interaction, both of the optical and mechanical resonance frequencies and damping rates undergo shifts. Even at the limit of zero input optical power $\alpha = 0$ and therefore zero cavity photon number $\bar{n} = 0$, it is possible to show that there is a temperature-dependent shift in the mechanical resonance frequency, markedly different from the lattice-expansion dependent effect. This effect is solely due to the optomechanical interaction with virtual cavity photons, which completely vanishes when $g_0 = 0$. In close relationship to the shift of resonances, we can also study the optomechanical spring effect with the corrections from higher-order interactions included.

The analysis of spring effect is normally done by consideration of the effective optomechanical force acting upon the damped mechanical oscillator, thus obtaining a shift in squared mechanical frequency $\delta(\Omega^2)$, whose real and imaginary parts give expressions for $\delta\Omega$ and $\delta\Gamma$. Corrections to these two terms due to higher-order interactions are discussed in the main article. Here, we demonstrate that the analysis using higher-order operator algebra can recover some important lost information regarding the optical and mechanical resonances when the analysis is done on the linearized basis $\{A\}^{\mathrm{T}} = \{\hat{a}, \hat{a}^\dagger, \hat{b}, \hat{b}^\dagger\}$.

To proceed, we consider finding eigenvalues of the matrix $\mathbf{M}$ as defined in (S19). Ignoring all higher-order nonlinear effects beyond the basis $\{A\}^{\mathrm{T}} = \{\hat{a}, \hat{a}\hat{b}, \hat{a}\hat{b}^\dagger\}$, we set $s = 0$. This enables us to search for the eigenvalues of the coefficients matrix $\mathbf{M}$ as

$$\begin{aligned}
\mathrm{eig}\{\mathbf{M}\} &= \mathrm{eig}\begin{bmatrix} i\Delta - \frac{\kappa}{2} & ig_0 & ig_0 \\ i(G+f^+) & -i(\Omega-\Delta) - \frac{\gamma}{2} & 0 \\ -i(G-f^-) & 0 & i(\Omega+\Delta) - \frac{\gamma}{2} \end{bmatrix} \\
&= i\left\{\begin{array}{c} \Delta + \lambda_1(\Delta,T) + i\gamma_1(\Delta,T) \\ \Delta + \lambda_2(\Delta,T) + i\gamma_2(\Delta,T) \\ \Delta + \lambda_3(\Delta,T) + i\gamma_3(\Delta,T) \end{array}\right\} \\
&= i\left\{\begin{array}{c} \Delta + \eta_1(\Delta,T) \\ \Delta + \eta_2(\Delta,T) \\ \Delta + \eta_3(\Delta,T) \end{array}\right\},
\end{aligned} \tag{S21}$$

in which $\lambda_j = \Re[\eta_j]$ and $\gamma_j = \Im[\eta_j]$ with $j = 1,2,3$ are real valued functions of Δ and bath temperature T. The temperature T determines $\bar{m}$ while $\bar{n}$ is a function of Δ as well as input photon rate α.

In general, the three eigenvalues $\eta_j = \lambda_j(\Delta,T) + i\gamma_j(\Delta,T)$, $j = 1,2,3$ are expected to be deviate from the three free-running values $\psi_1 = i\frac{1}{2}\kappa$, $\psi_2 = -\Omega + i\frac{1}{2}\gamma$, and $\psi_3 = \Omega + i\frac{1}{2}\gamma$, as $\eta_j \approx \psi_j - \Delta$ because of non-zero g_0. Solving the three equations therefore gives the values of shifted optical and mechanical frequencies and their damping rates compared to the bare values in absence of optomechanical interactions with $g_0 = 0$

$$\begin{aligned}
\delta\Omega &= -\frac{1}{2}\Re[\eta_2 - \eta_3] - \Omega, \\
\delta\omega &= -\frac{1}{2}\Re[\eta_2 + \eta_3], \\
\delta\Gamma &= \Im[-2\eta_1 + \eta_2 + \eta_3] - \Gamma, \\
\delta\kappa &= 2\Im[\eta_1] - \kappa.
\end{aligned} \tag{S22}$$

This method to calculate the alteration of resonances, does not regard the strength of the optomechanical interaction or any of the damping rates. In contrast, the known methods to analyze this phenomenon normally require $g << \kappa$ and $\Gamma + \delta\Gamma << \kappa$ [S2].

The above values can be calculated numerically for a typical optomechanical cavity, whose parameters are displayed in Table S1. The selected values of the four example optomechanical set ups result in very different configurations. System A is very strongly coupled with $g/(\kappa/2) = 1.2$ and in far Doppler limit $(\kappa/2)/\Omega = 15$. Meanwhile, Systems B and C with $(\kappa/2)/\Omega = 0.15$, and System D with $(\kappa/2)/\Omega = 2.2 \times 10^{-6}$ are all in the resolved-side band regime. System B is ultrastrongly coupled with $g/(\kappa/2) = 1.07 \times 10^4$, while for Systems C and D we have respectively $g/(\kappa/2) = 1.2$ and $g/(\kappa/2) = 3.48$.

A very simple way to estimate the shift in eigenvalues is by separating the real and imaginary parts of the optomechanical interaction, as $\Omega \to \Omega - \Re[s]$ and $\gamma \to \gamma + 2\Im[s]$. These shifts in mechanical frequency and damping rates can be approximated using (S6) as

$$\begin{aligned}
\delta\Omega + \delta\omega &\approx -g_0\Re[\bar{b}] = -\frac{g^2\Omega}{\Omega^2 + \frac{1}{4}\Gamma^2}, \\
\delta\Gamma + \delta\kappa &\approx g_0\Im[\bar{b}] = \frac{g^2\Gamma}{2(\Omega^2 + \frac{1}{4}\Gamma^2)},
\end{aligned} \tag{S23}$$

Table S1. Parameters of the simulated optomechanical example; $P_{\rm op}$: input optical power; λ: optical wavelength; Ω_m: mechanical angular frequency; Q: optical quality factor; Q_m: mechanical quality factor; g_0: single-photon optomechanical interaction rate; T: absolute temperature; $\mathscr{C}_0 = 4g_0^2/\kappa\Gamma$: single-photon cooperativity [S2]; $\mathscr{C} = \bar{n}\mathscr{C}_0$: multi-photon cooperativity. System A is strongly coupled and in Doppler regime. Systems B and C are side-band resolved but strongly coupled. System D is the one used in experiment [S11, S12]. System E is used for study of nonlinear transduction.

System	$P_{\rm op}$	λ	$\Omega_m/2\pi$	Q	Q_m	$g_0/2\pi$	T	$\mathscr{C}_0$	$\mathscr{C}$
A	2μW	1μm	1GHz	10^4	10^3	160kHz	1K	3.38×10^{-4}	3.81
B	2μW	1μm	1GHz	10^6	10^4	16kHz	1K	3.38×10^{-5}	3.41×10^3
C	20nW	1μm	1GHz	10^6	10^4	16kHz	1K	3.38×10^{-5}	0.381
D	450nW	1.55μm	5.3GHz	2.3×10^5	3.8×10^5	869kHz	35mK	1.5×10^{-2}	2.8×10^5
E	–	1μm	1GHz	10^6	10^4	400kHz	–	2.21×10^{-2}	–

where g has been defined under (S2). This approximation requires the optomechanical processes $\{\hat{a}\hat{b},\hat{a}\hat{b}^\dagger\}$ being independent of the other state variables. Since this decoupling is not exact, relations (S23) also will remain approximate. However, the accuracy of these are still quite remarkable as is demonstrated here.

First of all, it is noticed through extensive numerical computations that the shifts in optical and mechanical frequencies take place primarily in the optical part. That implies the resonance shift is typically much stronger in the optical partition of the system instead of the mechanical partition, leading to marked change in reflection spectra of optomechanical cavities. We calculate and plot each of the four individual components of (S22) along with the analytical expressions (S23) for the four systems A, B, C and D described in Table S1, respectively illustrated in Figs. S1, S2, S3 and S4 for the shifts in mechanical and optical frequencies and dissipation-decay rates.

Extensive numerical calculations for various configurations establish the fact that it is actually the optical resonance frequency which receives the optomechanical interaction effect. The asymmetry of this shift in cavity optical frequency across the zero-detuning $\Delta = 0$, is well exhibited in Fig. S2 for System B, and in Fig. S4 for System D, both of which are taken to have relatively large intracavity photon numbers around 10^7 to 10^8. This clear numerical signature underlines the fact that the well-known asymmetry of cavity optical response at high intensities should be actually a result of this higher-order spring effect, rather than thermally induced instabilities.

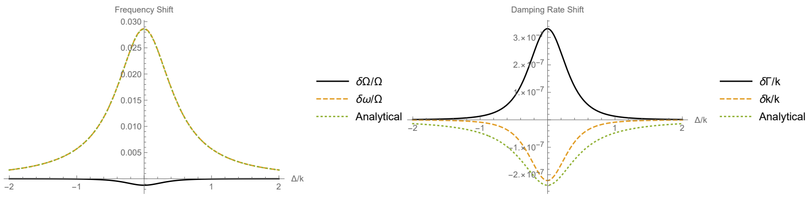

Figure S1. Shift in frequency and damping rates of optical and mechanical partitions due to optomechanical interaction for System A.

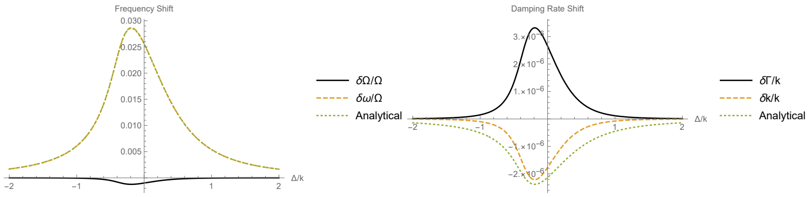

Figure S2. Shift in frequency and damping rates of optical and mechanical partitions due to optomechanical interaction for System B. Large cooperativity causes asymmetric behavior of frequency and damping shifts.

Summarizing, any such higher-order resonance shift will cause a change in mechanical frequency $\delta\Omega$ and decay rate $\delta\Gamma$, as well as optical detuning $\delta\omega = -\delta\Delta$ and decay rate $\delta\kappa$. While all these four components are non-zero, it is $\delta\Delta$ which is ultimately dominant over the three others in the bistability relation (S9). This will make the cavity response to follow the bistability and therefore appear to be asymmetric at high illumination drive intensities.

As a result of higher-order spring effect and $\delta\Delta$, a shift in intracavity photon number follows $\delta\bar{n}$, which immediately shifts

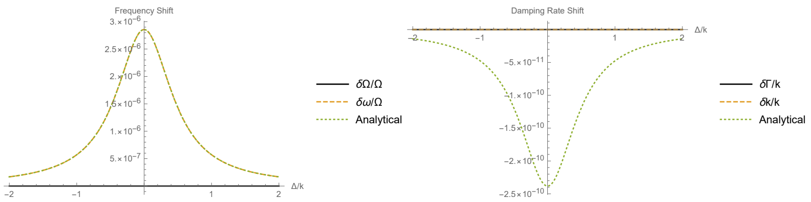

Figure S3. Shift in frequency and damping rates of optical and mechanical partitions due to optomechanical interaction for System C.

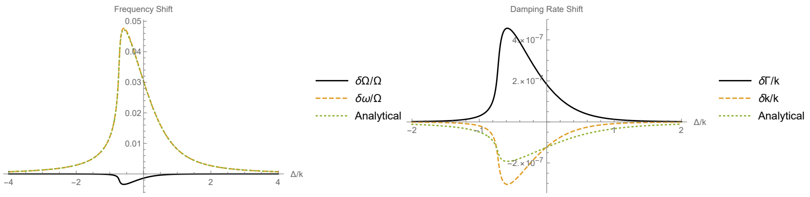

Figure S4. Shift in frequency and damping rates of optical and mechanical partitions due to optomechanical interaction for System D.

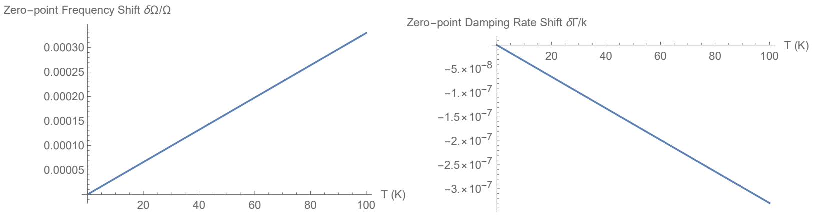

Figure S5. Temperature dependence of mechanical frequency shift due to optomechanical interaction with zero-point radiation field for System E. This temperature-dependent shift amounts to 3.3kHz/K.

the higher-order spring effect and therefore $\delta\Delta$. The infinite cycle of shifts in intracavity photon number and optical resonance frequency establishes a deterministic chaotic behavior, which is also a well known experimental observation in the community.

Furthermore, for System E, the zero-point optical field can change the mechanical frequency as large as 3.3kHz/K as illustrated in Fig. S5. Being in close relationship with the Dynamical Casimir effect [S13], this value could be in principle measured if temperature-induced expansion and the resulting change of mechanical frequency is much smaller. The thermal expansion coefficient of Silicon is roughly $2.6 \times 10^{-6}\text{K}^{-1}$, roughly equivalent to 2.6kHz/K. The contribution of zero-point field can be therefore larger or at least within the same order of magnitude. Here, we have assumed that the thermal expansion coefficient of Silicon is independent of temperature and also Ω shifts linearly with temperature.

The same calculation for System D gives out a value of 0.57kHz/K which is much less than the temperature expansion drift of 13.7kHz/K for the same structure. This phenomenon has been also noticed and referred to as the Nonlinear Transduction [S14] where the photon-phonon coupling can induce a temperature-dependent change in the resonance frequency of the cavity, even on the order of cavity linewidth.

S6 Coherent Phonon Population

It is here shown that the method of higher-order operators allows one to find an explicit expression for $\bar{m}$. In order to do this, we need to write down the 3×3 reduced set of higher-order optomechanical equations with the fluctuations terms dropped, which reads

$$\frac{d}{dt}\left\{\begin{array}{c} \hat{a} \\ \hat{a}\hat{b} \\ \hat{a}\hat{b}^\dagger \end{array}\right\} = \left[\begin{array}{ccc} i\Delta - \frac{1}{2}\kappa & ig_0 & ig_0 \\ ig_0(\bar{m}+\bar{n}+1) & -i(\Omega-\Delta)-\frac{1}{2}\gamma & 0 \\ ig_0(\bar{m}-\bar{n}) & 0 & i(\Omega+\Delta)-\frac{1}{2}\gamma \end{array}\right]\left\{\begin{array}{c} \hat{a} \\ \hat{a}\hat{b} \\ \hat{a}\hat{b}^\dagger \end{array}\right\} - \left\{\begin{array}{c} \alpha \\ \bar{b}\alpha \\ \bar{b}^*\alpha^* \end{array}\right\}. \tag{S24}$$

Here, α should be taken as a complex number from (S8), $\bar{b}$ is substituted from (S6) in terms of $\bar{a}$, where $\bar{a} = \sqrt{\bar{n}}$ is taken as a real number and $\bar{n}$ can already by found from the solution of the third-order algebraic equation (S9).

We are here interested in the steady state solutions, so that the time derivative $d/dt = 0$ can be set to zero. Then we arrive at the system of equations

$$\left[\begin{array}{ccc} i\Delta-\frac{1}{2}\kappa & ig_0 & ig_0 \\ ig_0(\bar{m}+\bar{n}+1) & -i(\Omega-\Delta)-\frac{1}{2}\gamma & 0 \\ ig_0(\bar{m}-\bar{n}) & 0 & i(\Omega+\Delta)-\frac{1}{2}\gamma \end{array}\right]\left\{\begin{array}{c} \bar{a} \\ \overline{ab} \\ \overline{ab^*} \end{array}\right\}=\left\{\begin{array}{c} \alpha \\ \bar{b}\alpha \\ \bar{b}^*\alpha^* \end{array}\right\}. \tag{S25}$$

In the above system of equations, $\overline{ab}$ corresponds to the time-average of the operators $\langle\hat{a}\hat{b}\rangle$, while $\overline{ab^*}$ corresponds to the time-average of the operators $\langle\hat{a}\hat{b}^\dagger\rangle$. Quite obviously, $\overline{ab}\approx\bar{a}\bar{b}$ and $\overline{ab^*}\approx\bar{a}\bar{b}^*$ can approximately hold based on the mean-field approximation. We shall here furthermore observe that this approximation does not any longer hold for the coherent phonons as $\bar{m}=\langle\hat{b}^\dagger\hat{b}\rangle\neq\bar{b}^*\bar{b}$ for the reasons discussed below.

We now can rearrange (S25) in terms of the unknown quantities $\bar{m}$, $\overline{ab}$, and $\overline{ab^*}$ as

$$\left[\begin{array}{ccc} 0 & ig_0 & ig_0 \\ ig_0\bar{a} & -i(\Omega-\Delta)-\frac{1}{2}\gamma & 0 \\ ig_0\bar{a} & 0 & i(\Omega+\Delta)-\frac{1}{2}\gamma \end{array}\right]\left\{\begin{array}{c} \bar{m} \\ \overline{ab} \\ \overline{ab^*} \end{array}\right\}=\left\{\begin{array}{c} \alpha-\left(i\Delta-\frac{1}{2}\kappa\right)\bar{a} \\ \bar{b}\alpha-ig_0\left(\bar{n}+1\right)\bar{a} \\ \bar{b}^*\alpha^*+ig_0\bar{n}\bar{a} \end{array}\right\}. \tag{S26}$$

This linear system of equations after appropriate substitutions from (S6) and (S8) now can be solved to find

$$\bar{m}(\Delta)=\frac{64g_0^2\Omega^2\bar{n}^2(\Delta)\left(\gamma^2+\gamma\Gamma+4\Delta^2\right)-\left[\left(\Gamma^2+4\Omega^2\right)^2\left(\gamma^2+4\Delta(\Delta+\Omega)\right]}{2\left(\gamma^2+4\Delta^2\right)\left(\Gamma^2+4\Omega^2\right)^2}. \tag{S27}$$

Here, a small imaginary part remains which has to be dropped and results from the inexactness of (S6) and (S8) coming from linearized optomechanics, and not being in complete consistency with the higher-order formalism.

In a similar manner, one may find

$$\begin{aligned}\overline{ab} &= \frac{i\sqrt{\bar{n}}\left[g_0^2\left(8\bar{n}+4\right)+(2\Delta+i\kappa)(2(\Delta+\Omega)+i\gamma)\right]-2i\alpha\left[\frac{8\Gamma g_0^2\bar{n}}{\Gamma^2+4\Omega^2}+\gamma-2i(\Delta+\Omega)\right]}{4g_0(\gamma-2i\Delta)}, \\ \overline{ab^*} &= \frac{2\alpha\left[\frac{8i\Gamma g_0^2\bar{n}}{\Gamma^2+4\Omega^2}-i\gamma-2\Delta+2\Omega\right]-\sqrt{\bar{n}}\left[4ig_0^2\left(2\bar{n}+1\right)+(\kappa-2i\Delta)(i\gamma+2\Delta-2\Omega)\right]}{4g_0(\gamma-2i\Delta)}.\end{aligned} \tag{S28}$$

The expression (S27) for $\bar{m}$ is accurate within half a quanta $\pm\frac{1}{2}$, so that in order to satisfy the zero limits at infinite detuning

$$\lim_{\Delta\to\infty}\bar{m}(\Delta)=0, \tag{S29}$$

a half-quanta must be added to (S27). Then it will read

$$\begin{aligned}\bar{m}(\Delta) &= \frac{32g_0^2\Omega^2\left(\gamma^2+\gamma\Gamma+4\Delta^2\right)}{\left(\gamma^2+4\Delta^2\right)\left(\Gamma^2+4\Omega^2\right)^2}\bar{n}^2(\Delta)-\frac{2\Delta\Omega}{\gamma^2+4\Delta^2}\pm\frac{1}{2} \\ &\approx \frac{32g_0^2\Omega^2\left(\gamma^2+\gamma\Gamma+4\Delta^2\right)}{\left(\gamma^2+4\Delta^2\right)\left(\Gamma^2+4\Omega^2\right)^2}\bar{n}^2(\Delta) \\ &= g_0^2\zeta(\Delta)\bar{n}^2(\Delta).\end{aligned} \tag{S30}$$

The approximation holds well if $\bar{n}$ is well above unity. Hence, we can infer from (S30) that $\bar{m}\propto\bar{n}^2$. In the lossless limit, where $\gamma\approx 0$ and $\Gamma\approx 0$, one may even further simplify (S30) to obtain the simple expression $\bar{m}\approx 2g_0^2\bar{n}^2/\Omega^2$ which is typically accurate within 10% of the actual value or better. It is not difficult to check the resonant coherent phonon number $\bar{m}(0)$. In the practical limit of $\kappa>>\Gamma$, it is easy to verify that (S30) actually simplifies to $\bar{m}(0)\approx 32[g_0 Q_{\rm m}\bar{n}(0)/\Gamma]^2$ with $Q_{\rm m}=\Omega/\Gamma$ being the mechanical quality factor. In the next section, we point out a straightforward method to measure this quantity through experiment on the well-known optical spring effect.

In practice, the expression (S30) is sensitive to the choice of optomechanical parameters and in particular g_0. A slight variation in the basic optomechanical parameters $\{g_0,\omega,\kappa,\Omega,\Gamma\}$ with $\gamma=\kappa+\Gamma$ as small as few percent can make a pronounced effect in expected behavior in $\bar{m}$.

For the side-band resolved systems in the lossless limit, it is within 10% of the relationship $\bar{m}\propto 2|\bar{b}|^2$, meanwhile for Doppler cavities, the agreement is roughly within 3% or better. This result perfectly agrees to the large-amplitude oscillation limit of $\bar{b}(t)\approx\delta\hat{b}(t)+[\bar{b}+\bar{b}\exp(-i\Omega t)]$ where $\delta\hat{b}$ represents the random fluctuations in the mechanical field with $\langle\delta\hat{b}\rangle=0$.

This also tells that the coherent oscillations of the mechanical field are not differential in amplitude, and can vary in the range $(0, 2|\bar{b}|)$. So, the amplitude of coherent mechanical oscillations is just as big as their average. This large-amplitude coherent mechanical wave is driven and waked by the optical coherent field inside the cavity, through optomechanical interactions. The mean field approximations $\overline{ab} \approx \bar{a}\bar{b}$ and $\overline{ab^*} \approx \bar{a}\bar{b}^*$ seem however to always hold better than 0.1% for Doppler cavities. This accuracy breaks down for side-band resolved cavities.

If there are more than one mechanical fields available $j = 1, 2, \cdots$, the coherent phonon population of each mode $\bar{m}_j$ shall be determined with the corresponding sets of optomechanical parameters $\left\{g_{0,j}, \omega_j, \kappa_j, \Omega_j, \Gamma_j\right\}$, with the expected approximate result $\bar{m}_j \approx 2g_{0,j}^2 \bar{n}_j^2/\Omega_j^2$ as long as the mechanical modes are almost uncorrelated. The case of coherent phonon numbers of two or more correlated mechanical modes needs a separate study.

It is here again stressed out that the oscillations of the mechanical field can be decomposed into the incoherent and coherent parts. The incoherent part results from random thermal fluctuations with the thermal occupancy m, as well as half a quanta contributing from the quantum noise of the coherent part, while the coherent oscillations correspond to the coherent phonon number $\bar{m}$. The same also should be true for the optical field, however, the random fluctuations of a coherent light is only half a quanta, and the thermal optical occupancy n of optomechanical cavity is normally negligible under practical considerations and working temperatures.

S7 Higher-order Spring Effect

It is possible to calculate the optomechanical spring effect due to the standard linearized and higher-order interactions. In order to do this, we start from the matrix $[\mathbf{M}]$ given in (S19), and after dropping the noise and drive input terms we notice the expansion of first Langevin equation for the operator $\hat{a}$. That reads

$$\frac{d}{dt}\hat{a} = (i\Delta - \frac{1}{2}\kappa)\hat{a} + ig_0\hat{a}(\hat{b} + \hat{b}^\dagger). \tag{S31}$$

From the second and third equations we get

$$\begin{aligned} \hat{a}\frac{d}{dt}\hat{b} + \left[(i\Delta - \frac{1}{2}\kappa)\hat{a} + ig_0\hat{a}(\hat{b} + \hat{b}^\dagger)\right]\hat{b} &= i(f^+ + F)\hat{a} - [i(\Omega - \Delta - g_0\hat{b}) + \frac{1}{2}\gamma]\hat{a}\hat{b}, \\ \hat{a}\frac{d}{dt}\hat{b}^\dagger + \left[(i\Delta - \frac{1}{2}\kappa)\hat{a} + ig_0\hat{a}(\hat{b} + \hat{b}^\dagger)\right]\hat{b}^\dagger &= i(f^- - F)\hat{a} + [i(\Omega + \Delta + g_0\hat{b}) - \frac{1}{2}\gamma]\hat{a}\hat{b}^\dagger. \end{aligned} \tag{S32}$$

This is equivalent to

$$\begin{aligned} \frac{d}{dt}\hat{b} &= i(f^+ + F + \hat{b}^\dagger\hat{b}) - (i\Omega + \frac{1}{2}\Gamma)\hat{b}, \\ \frac{d}{dt}\hat{b}^\dagger &= i(f^- - F + \hat{b}\hat{b}^\dagger) + (i\Omega - \frac{1}{2}\Gamma)\hat{b}^\dagger. \end{aligned} \tag{S33}$$

These two equations can be now combined after dropping the nonlinear terms by addition and subtraction, and then taking the Fourier transform to yield the system

$$\begin{bmatrix} -iw + \frac{1}{2}\Gamma & i\Omega \\ i\Omega & -iw + \frac{1}{2}\Gamma \end{bmatrix} \begin{Bmatrix} \hat{b} + \hat{b}^\dagger \\ \hat{b} - \hat{b}^\dagger \end{Bmatrix} = 2ig_0 \begin{Bmatrix} \bar{m} + \frac{1}{2} \\ \bar{n} + \frac{\Gamma}{2} \end{Bmatrix}. \tag{S34}$$

This can be solved now to yield the expression for $\delta\hat{x} = x_{\text{zp}}(\hat{b} + \hat{b}^\dagger)$ as

$$\delta\hat{x}(w) = 2ix_{\text{zp}}g_0 \frac{(-iw + \frac{1}{2}\Gamma)(\bar{m} + \frac{1}{2}) - i\Omega(\bar{n} + \frac{1}{2})}{(-iw + \frac{1}{2}\Gamma)^2 - (-i\Omega)^2}. \tag{S35}$$

A rearrangement of this expression yields

$$\delta\hat{x}(w) = \frac{2x_{\text{zp}}g_0\Omega}{-(w + i\frac{1}{2}\Gamma)^2 + \Omega^2} \left\{\bar{n} + \left[\left(\frac{w}{\Omega} + i\frac{\Gamma}{2\Omega}\right)\left(\bar{m} + \frac{1}{2}\right) + \frac{1}{2}\right]\right\}. \tag{S36}$$

It is straightforward now to see that the term within brackets contributes to the necessary corrections to the spring effect. This will change the mechanical response function $\Sigma(w)$ [S1, S2, S4, S15] as

$$\Sigma(w, \Delta) = 2\Omega g_0^2 \left[\frac{1}{(\Delta + w) + \frac{i}{2}\kappa} + \frac{1}{(\Delta - w) - \frac{i}{2}\kappa}\right][\bar{n} + \mu(w)], \tag{S37}$$

where a term with the dimension of mass in the numerator, which in the following calculation ultimately cancels out, and is equal to the effective motion mass $m_{\rm eff}$, is not shown for simplicity. We have also

$$\mu(w) = \frac{1}{\Omega}\left(w + \frac{i}{2}\Gamma\right)\left(\bar{m} + \frac{1}{2}\right) + \frac{1}{2}, \tag{S38}$$

represents corrections to yield the effective cavity photon number $\bar{n}_{\rm eff} = \bar{n} + \mu(w)$ because of higher-order interactions. This corrections is easy to see that are important if the pump level is not too high. Typically, for $\bar{n} < 10^2$ higher-order spring effects are quite significant, and when $\bar{n} > 10^3$ the higher-order effects are suppressed by the standard spring effect.

The spring effect modifies the measured mechanical frequency Ω and linewidth Γ as

$$\begin{aligned} \delta\Omega(w,\Delta) &= \frac{1}{2w}\Re[\Sigma(w,\Delta)], \\ \delta\Gamma(w,\Delta) &= -\frac{1}{w}\Im[\Sigma(w,\Delta)]. \end{aligned} \tag{S39}$$

Put together combined, we get

$$\begin{aligned} \delta\Omega(w,\Delta) &= \frac{g_0^2\bar{n}\Omega}{w}\left[\frac{\Delta+w}{(\Delta+w)^2+\frac{1}{4}\kappa^2} + \frac{\Delta-w}{(\Delta-w)^2+\frac{1}{4}\kappa^2}\right] \\ &+ \frac{g_0^2\Re[\mu(w)]\Omega}{w}\left[\frac{\Delta+w}{(\Delta+w)^2+\frac{1}{4}\kappa^2} + \frac{\Delta-w}{(\Delta-w)^2+\frac{1}{4}\kappa^2}\right] \\ &+ \frac{g_0^2\Im[\mu(w)]\Omega}{w}\left[\frac{\kappa}{(\Delta+w)^2+\frac{1}{4}\kappa^2} - \frac{\kappa}{(\Delta-w)^2+\frac{1}{4}\kappa^2}\right], \\ \delta\Gamma(w,\Delta) &= \frac{g_0^2\bar{n}\Omega}{w}\left[\frac{\kappa}{(\Delta+w)^2+\frac{1}{4}\kappa^2} - \frac{\kappa}{(\Delta-w)^2+\frac{1}{4}\kappa^2}\right] \\ &+ \frac{g_0^2\Re[\mu(w)]\Omega}{w}\left[\frac{\kappa}{(\Delta+w)^2+\frac{1}{4}\kappa^2} - \frac{\kappa}{(\Delta-w)^2+\frac{1}{4}\kappa^2}\right] \\ &- \frac{g_0^2\Im[\mu(w)]\Omega}{w}\left[\frac{\Delta+w}{(\Delta+w)^2+\frac{1}{4}\kappa^2} + \frac{\Delta-w}{(\Delta-w)^2+\frac{1}{4}\kappa^2}\right]. \end{aligned} \tag{S40}$$

Here, the second and third terms on the rights hand sides of both equations are corrections to the spring effect due to the higher-order interactions, resulting from the temperature-dependent expressions

$$\begin{aligned} \Re[\mu(w)] &= \frac{w}{\Omega}\left(\bar{m}+\frac{1}{2}\right)+\frac{1}{2}, \\ \Im[\mu(w)] &= \frac{\Gamma}{2\Omega}\left(\bar{m}+\frac{1}{2}\right). \end{aligned} \tag{S41}$$

The temperature-dependence of (S41) causes dependence of the spring effect on temperature as well.

The uncorrected standard expressions read [S2, S15]

$$\begin{aligned} \delta\Omega(w,\Delta) &= \frac{g_0^2\bar{n}\Omega}{w}\left[\frac{\Delta+w}{(\Delta+w)^2+\frac{1}{4}\kappa^2} + \frac{\Delta-w}{(\Delta-w)^2+\frac{1}{4}\kappa^2}\right], \\ \delta\Gamma(w,\Delta) &= \frac{g_0^2\bar{n}\Omega}{w}\left[\frac{\kappa}{(\Delta+w)^2+\frac{1}{4}\kappa^2} - \frac{\kappa}{(\Delta-w)^2+\frac{1}{4}\kappa^2}\right], \end{aligned} \tag{S42}$$

from which we may observe

$$\begin{aligned} \delta\Omega(w,\Delta) &= -\delta\Omega(w,-\Delta), \\ \delta\Omega(-\Delta,\Delta) &= \frac{g_0^2\bar{n}\Omega}{2}\frac{\Delta}{\Delta^2+\frac{1}{16}\kappa^2}, \end{aligned} \tag{S43}$$

which do hold for the standard spring effect at sufficiently high optical powers.

S7.1 Examples

As an example, we first assume a side-band resolved cavity with optical resonance wavelength of $\lambda = 1\mu$m and quality factor $Q = 10^6$, a mechanical resonance frequency of $\Omega = 2\pi \times 1$GHz and quality factor of $Q_\text{m} = 10^5$, with the optomechanical interaction rate $g_0 = 2\pi \times 160$kHz. Pumping resonantly at the rates $\alpha = 10^{10}\text{s}^{-1}$ and $\alpha = 10^{11}\text{s}^{-1}$ correspond to $\bar{n} = 1.1 \times 10^2$ and $\bar{n} = 1.1 \times 10^4$.

In Figs. S6 and S7, optical spring effect has been illustrated at the two above different pumping rates, respectively, for a side-band resolved cavity. It has been supposed that a two-beam pump-probe experiment is undertaken, where the pump frequency is having the detuning Δ and the relative probe frequency is w.

It can be seen that when the cavity photon number $\bar{n}$ is increased while coherent phonon number $\bar{m}$ is kept constant, the higher-order corrections to the spring effect are just negligible and can be ignored, as shown in Fig. S7. However, at lower pumping rates where $\bar{n}$ is no longer much larger than $\bar{m}$, the higher-order corrections become important as shown in Fig. S6. Ultimately, for a few or very low cavity photon number $\bar{n} < 10$, the higher-order corrections could be orders of magnitude stronger than the standard effects.

In Fig. S8 the same cavity parameters are employed with a lowered optical quality factor of $Q = 10^4$. This choice puts the cavity in the Doppler regime. It is easy to see again that in the same manner of the previous examples, sufficiently high pump levels entirely masks the higher-order effects. Taking only the case of $\alpha = 3 \times 10^{11}\text{s}^{-1}$ here leads to $\bar{n} = 1.01 \times 10^1$, while at smaller pump levels only the higher-order optomechanical effects survive. At this cavity photon number around $\bar{n} \approx 10$, differences between the standard and higher-order corrected responses are quite prominent and visible.

S8 Minimal Basis

Complete solution of optomechanical interaction $\mathbb{H}_\text{OM}$ can be attained analytically using the minimal basis $\{A\}^\text{T} = \{\hat{n}^2, \hat{n}\hat{b}, \hat{n}\hat{b}^\dagger\} = \{\hat{N}, \hat{B}, \hat{B}^\dagger\}$. Construction of Langevin equations leads to the system

$$\frac{d}{dt}\left\{\begin{array}{c}\hat{N}\\ \hat{B}\\ \hat{B}^\dagger\end{array}\right\} = \left[\begin{array}{ccc}-2\kappa & 0 & 0\\ ig_0 & -i\Omega-\frac{\gamma}{2} & 0\\ ig_0 & 0 & i\Omega-\frac{\gamma}{2}\end{array}\right]\left\{\begin{array}{c}\hat{N}\\ \hat{B}\\ \hat{B}^\dagger\end{array}\right\} - \left\{\begin{array}{c}\sqrt{4\kappa}\hat{N}_\text{in}\\ \sqrt{\gamma}\hat{B}_\text{in}\\ \sqrt{\gamma}\hat{B}_\text{in}^\dagger\end{array}\right\}. \tag{S44}$$

Here, the multiplicative noise terms are defined as

$$\begin{aligned}\sqrt{4\kappa}\hat{N}_\text{in} &= 2\sqrt{\kappa}\left(\hat{n}\hat{a}^\dagger\hat{a}_\text{in} + \hat{a}_\text{in}^\dagger\hat{a}\hat{n}\right), \\ \sqrt{\gamma}\hat{B}_\text{in} &= \sqrt{2\kappa}\hat{b}\hat{n}_\text{in} + \sqrt{\Gamma}\hat{n}\hat{b}_\text{in},\end{aligned} \tag{S45}$$

where $\hat{n}_\text{in}$ is already defined under (S1), and the spectral density of which can be estimated using the method described elsewhere [S16]. A very effective method to deal with multiplicative noise is to be discussed in §S10. This can be immediately noticed to be reducible as

$$\frac{d}{dt}\left\{\begin{array}{c}\hat{N}\\ \hat{B}\end{array}\right\} = \left[\begin{array}{cc}-2\kappa & 0\\ ig_0 & -i\Omega-\frac{\gamma}{2}\end{array}\right]\left\{\begin{array}{c}\hat{N}\\ \hat{B}\end{array}\right\} - \left\{\begin{array}{c}\sqrt{4\kappa}\hat{N}_\text{in}\\ \sqrt{\gamma}\hat{B}_\text{in}\end{array}\right\}. \tag{S46}$$

These will make the evaluation of spectral densities $S_{NN}(\omega)$ and $S_{BB}(\omega)$ possible. Interestingly, (S46) is actually decoupled, since the equation for $\hat{N}$ is already independent of $\hat{B}$, which admits the solution

$$\hat{N}(t) = \hat{N}(0)e^{-2\kappa t} - 2\sqrt{\kappa}e^{-2\kappa t}\int_0^t \hat{N}_\text{in}(\tau)e^{2\kappa\tau}d\tau. \tag{S47}$$

We can be now plug (S47) in the second equation of (S46) to solve exactly for $\hat{B}$. We define $\vartheta = i\Omega + \frac{\gamma}{2}$ and may write down

$$\hat{B}(t) = \hat{B}(0)e^{-\vartheta t} - e^{-\vartheta t}\int_0^t e^{\vartheta\tau}\left[ig_0\hat{N}(\tau) + \sqrt{\gamma}\hat{B}_\text{in}(\tau)\right]d\tau. \tag{S48}$$

The treatment of multiplicative noise terms (S45) can be quite difficult in the most general form, especially that they demand prior knowledge of photonic and phononic ladder operators. However, assuming that the extra ladder operators can be replaced by their mean values, we can do the zeroth order approximations

$$\begin{aligned}\sqrt{4\kappa}\hat{N}_\text{in} &\approx \sqrt{\kappa\bar{n}}\bar{n}\left(\hat{a}_\text{in} + \hat{a}_\text{in}^\dagger\right) \to 2\sqrt{\kappa\bar{n}}\bar{n}\breve{a}_\text{in}, \\ \sqrt{\gamma}\hat{B}_\text{in} &\approx \sqrt{\kappa\bar{n}}\bar{b}\left(\hat{a}_\text{in} + \hat{a}_\text{in}^\dagger\right) + \sqrt{\Gamma}\bar{n}\hat{b}_\text{in} \to 2\sqrt{\kappa\bar{n}}\bar{b}\breve{a}_\text{in} + \sqrt{\Gamma}\bar{n}\hat{b}_\text{in}.\end{aligned} \tag{S49}$$

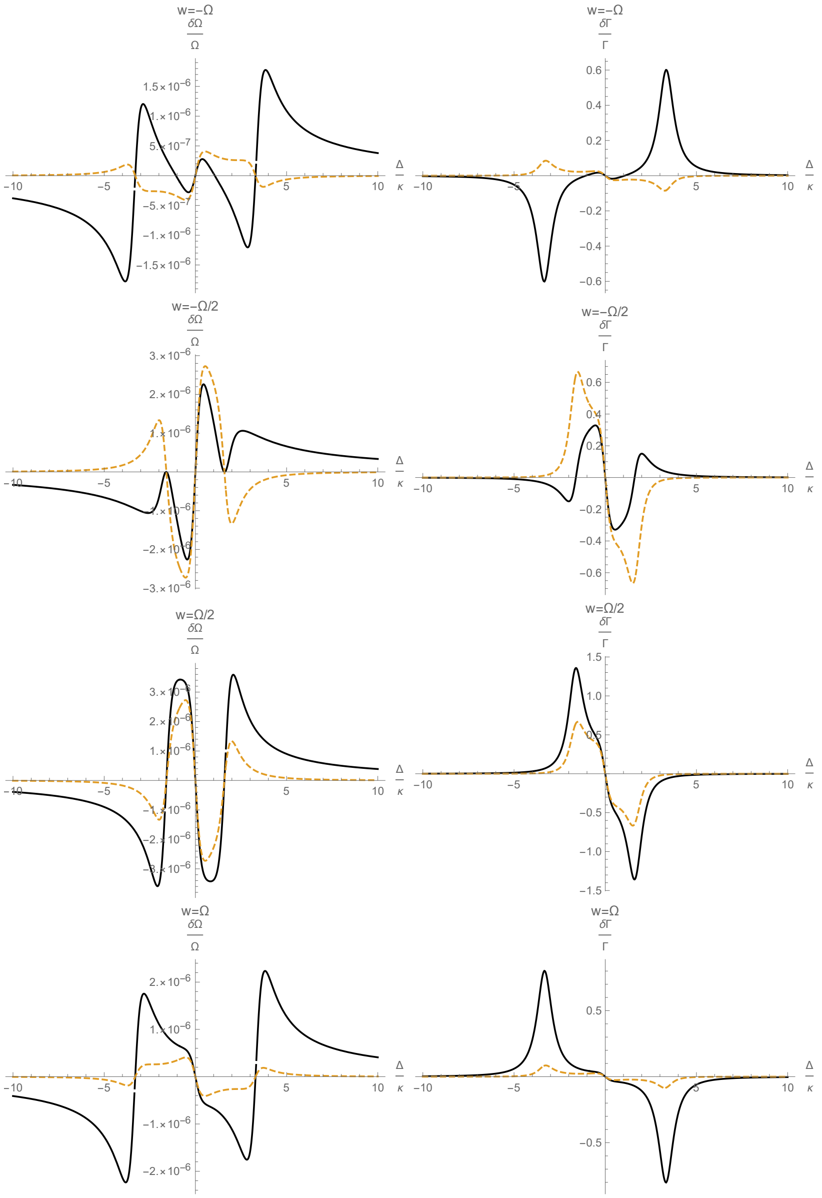

Figure S6. Optical spring effect due to the standard (dashed) and higher-order interactions (solid black) for a two-beam measurement. From top to the bottom: $w = -\Omega$, $w = -\frac{1}{2}\Omega$, $w = \frac{1}{2}\Omega$, and $w = -\Omega$. Left column corresponds to the change in mechanical frequency $\delta\Omega$ while the right column corresponds to the change in linewidth $\delta\Gamma$. This cavity is side-band resolved and $\alpha = 10^{10}\mathrm{s}^{-1}$.

Here, the real-valued Weiner process $\breve{a}_{\mathrm{in}}(t)$ with the *symmetrized* classical spectral density $\breve{a}_{\mathrm{in}}(\omega)$ is obtained as

$$\begin{aligned} \breve{a}_{\mathrm{in}}(t) &= \frac{\hat{a}_{\mathrm{in}}(t) + \hat{a}_{\mathrm{in}}^{\dagger}(t)}{2}, \\ \breve{a}_{\mathrm{in}}(\omega) &= \Re[\hat{a}_{\mathrm{in}}(\omega)]. \end{aligned} \tag{S50}$$

While this type of approximations in multiplicative noise could be useful for many cases, there are some phenomena which cannot be reproduced without correct treatment of multiplicative noise. This shall be discussed in details in §S10. Nevertheless, it is also instructive the take the expectation values of (S44) to obtain the classical system

$$\frac{d}{dt}\left\{\begin{array}{c} N(t) \\ B(t) \end{array}\right\} = \left[\begin{array}{cc} -2\kappa & 0 \\ ig_0 & -i\Omega - \frac{\gamma}{2} \end{array}\right]\left\{\begin{array}{c} N(t) \\ B(t) \end{array}\right\} + 2\sqrt{\bar{n}}\left\{\begin{array}{c} \bar{n} \\ \bar{b} \end{array}\right\}\Re[\alpha]. \tag{S51}$$

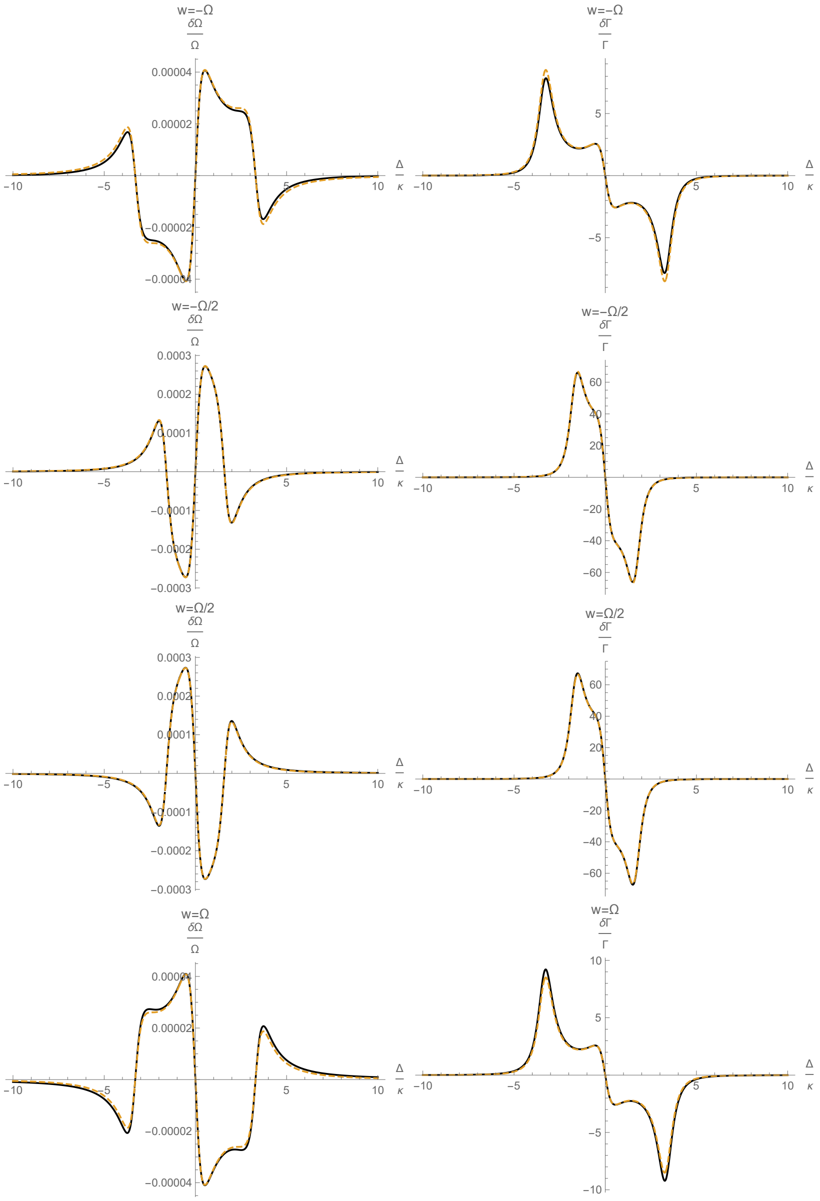

Figure S7. Optical spring effect due to the standard (dashed) and higher-order interactions (solid black) for a two-beam measurement. From top to the bottom: $w = -\Omega$, $w = -\frac{1}{2}\Omega$, $w = \frac{1}{2}\Omega$, and $w = -\Omega$. Left column corresponds to the change in mechanical frequency $\delta\Omega$ while the right column corresponds to the change in linewidth $\delta\Gamma$. This cavity is side-band resolved and $\alpha = 10^{11}\text{s}^{-1}$.

Together with (S6,S9), and setting the time-derivative on the left of the above to zero, makes the evaluation of steady-state values $N(\infty) = \bar{n}^2$ and $B(\infty) = \overline{nb} \approx \bar{n}\bar{b}$ readily possible. Doing this and solving for $\bar{n}$ and $\bar{b}$ precisely gives back (S6). This not only is in agreement with the equilibrium equation (S9), but also confirms the general finding that the equilibrium intracavity photon population $\bar{n}(\Delta)$ is independent of the coherent phonon population $\bar{m}(\Delta)$. However, the opposite is not correct, and as it was shown in the previous sections, $\bar{m}(\Delta)$ can actually be either determined from $\bar{n}(\Delta)$ and fitting to the experimental data, or directly estimated using the expression (S30) in §S6.

Existence of such an exact transformation which puts the optomechanical interaction into exactly linear form should be connected to the polaron transformation [S2] which leaves behind a Kerr nonlinear term as $\hat{n}^2$ in the transformed optomechanical Hamiltonian. It furthermore highlights the fact that usage of higher-order operators ultimately can reach a fully linear system at which convergence of this method to the exact solution is evident.

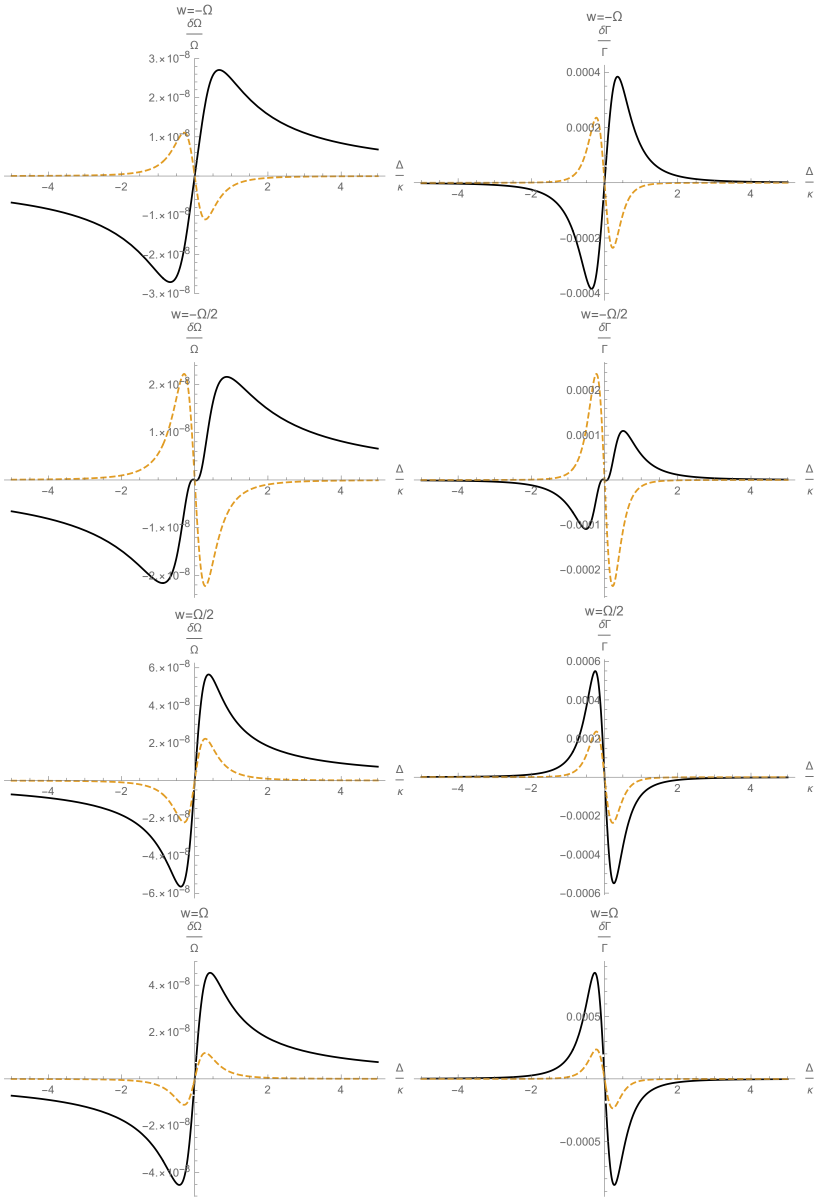

Figure S8. Optical spring effect due to the standard (dashed) and higher-order interactions (solid black) for a two-beam measurement. From top to the bottom: $w = -\Omega$, $w = -\frac{1}{2}\Omega$, $w = \frac{1}{2}\Omega$, and $w = -\Omega$. Left column corresponds to the change in mechanical frequency $\delta\Omega$ while the right column corresponds to the change in linewidth $\delta\Gamma$. This cavity is in strong Doppler regime and $\alpha = 3 \times 10^{11}\mathrm{s}^{-1}$.

S9 Higher-Order Sidebands

When the optical frequency is much larger than the mechanical frequency, apart from the mechanical sidebands which are roughly placed at $\Delta^{(1)} \approx \pm\Omega$, there exist higher-order sidebands such as $\Delta^{(2)} \approx \pm 2\Omega$ and so on. The occurrence of these higher-order sidebands, which are observable for sideband-resolved experiments, is obviously stringent on the existence of two- and multi-phonon processes. Normally, one would expect that these could be studied by constructing the Langevin equations for the operators $\hat{b}^2$, $\hat{b}^{\dagger 2}$ and so on. But this guess turns out to be incorrect, since the corresponding Langevin equations would be totally independent of the one for $\hat{a}$, implying that the second- and higher-order sidebands could not be reconstructed via the fully linearized Langevin equations. This has already been shown to be a nonlinear process which does not naturally appear in the Hamiltonian of the fully linearized optomechanics [S17]. But this difficulty can be appropriately addressed by the method of Higher-order Operators, too.

In order to investigate this phenomenon, let us restrict the case only to the second-order sidebands roughly located at

$\Delta^{(2)} \approx \pm 2\Omega$. In order to study these, it is sufficient to extend the basis $\{A\}^{\mathrm{T}} = \left\{\hat{a}, \hat{a}\hat{b}, \hat{a}\hat{b}^{\dagger}\right\}$ to

$$\{A\}^{\mathrm{T}} = \{\hat{a}, \hat{a}\hat{b}, \hat{a}\hat{b}^{\dagger}, \hat{a}\hat{b}^{2}, \hat{a}\hat{b}^{\dagger 2}\}, \tag{S52}$$

where the third-rank higher-order operators $\hat{a}\hat{b}^2$ and $\hat{a}\hat{b}^{\dagger 2}$ take care of the one-photon two-phonon processes, ultimately leading to formation of second-order sidebands at $\Delta^{(2)} \approx \pm 2\Omega$. The Langevin equations for this basis within the zeroth-order approximation of multiplicative noise reads

$$\left[\begin{array}{ccc|cc} i\Delta - \frac{\kappa}{2} & ig_0 & ig_0 & 0 & 0 \\ ig_0(\bar{m}+\bar{n}+1) & -i(\Omega-\Delta)-\frac{\gamma}{2} & 0 & ig_0 & 0 \\ ig_0(\bar{m}-\bar{n}) & 0 & i(\Omega+\Delta)-\frac{\gamma}{2} & 0 & ig_0 \\ \hline 0 & ig_0(\bar{m}+2\bar{n}+2) & 0 & -i(2\Omega-\Delta)-\frac{\theta}{2} & 0 \\ 0 & 0 & ig_0(\bar{m}-2\bar{n}-1) & 0 & i(2\Omega+\Delta)-\frac{\theta}{2} \end{array}\right]$$
$$\times \left\{\begin{array}{c} \hat{a} \\ \hat{a}\hat{b} \\ \hat{a}\hat{b}^{\dagger} \\ \hline \hat{a}\hat{b}^{2} \\ \hat{a}\hat{b}^{\dagger 2} \end{array}\right\} - \left[\begin{array}{ccc} \sqrt{\kappa} & 0 & 0 \\ \sqrt{\frac{1}{2}\kappa\bar{m}} & \sqrt{\Gamma\bar{n}} & 0 \\ \sqrt{\frac{1}{2}\kappa\bar{m}} & 0 & \sqrt{\Gamma\bar{n}} \\ \hline \frac{1}{2}\sqrt{\kappa\bar{m}} & \sqrt{\Gamma\bar{n}\bar{m}} & 0 \\ \frac{1}{2}\sqrt{\kappa\bar{m}} & 0 & \sqrt{\Gamma\bar{n}\bar{m}} \end{array}\right] \left\{\begin{array}{c} \hat{a}_{\mathrm{in}} \\ \hat{b}_{\mathrm{in}} \\ \hat{b}_{\mathrm{in}}^{\dagger} \end{array}\right\} + \left[\begin{array}{cc} 1 & 0 \\ \bar{b} & 0 \\ \bar{b}^{*} & 0 \\ \hline \bar{b}^{2} & 0 \\ \bar{b}^{*2} & 0 \end{array}\right] \left\{\begin{array}{c} \alpha \\ \alpha^{*} \end{array}\right\} = \frac{d}{dt}\left\{\begin{array}{c} \hat{a} \\ \hat{a}\hat{b} \\ \hat{a}\hat{b}^{\dagger} \\ \hline \hat{a}\hat{b}^{2} \\ \hat{a}\hat{b}^{\dagger 2} \end{array}\right\}. \tag{S53}$$

Here, $\theta = \kappa + 2\Gamma$ is the decay rate associated with the third-rank one-photon two-phonon processes $\hat{a}\hat{b}^2$ and $\hat{a}\hat{b}^{\dagger 2}$. The approximation $2|\bar{b}|^2 \approx \bar{m}$ is used following the discussions in §S6. We do observe that this treatment of multiplicative noise causes non-negligible error in some cases, and is due to be discussed later in §S10.

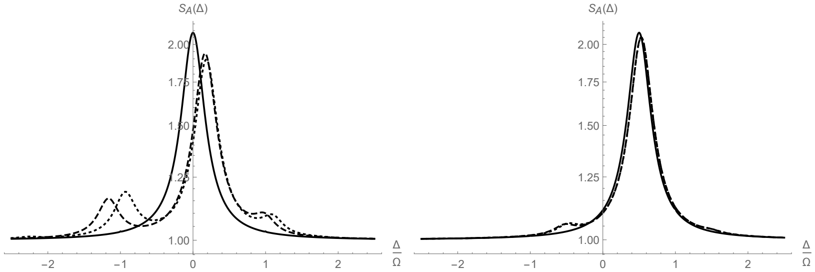

Figure S9. Estimated noise spectrum of a side-band resolved optomechanical cavity under strong pump according to the fully linearized optomechanics shown in solid black, and higher-order optomechanics with the bases $\{A\}^{\mathrm{T}} = \left\{\hat{a}, \hat{a}\hat{b}, \hat{a}\hat{b}^{\dagger}\right\}$ and (S52) respectively shown in dashed and dotted curves: resonant pump (left); pump on the red mechanical sideband (right).

Vertical and horizontal partitions separate the single-phonon $\{\hat{a}\hat{b}, \hat{a}\hat{b}^{\dagger}\}$ and two-phonon processes $\{\hat{a}\hat{b}^2, \hat{a}\hat{b}^{\dagger^2}\}$. So by retaining only the first 3×3 blocks and first 3 rows what remains is nothing but the equations of first-order optomechanics in terms of the second-rank single-phonon operator basis $\{\hat{a}, \hat{a}\hat{b}, \hat{a}\hat{b}^{\dagger}\}$.

The first-order $\delta\Delta^{(1)}$ and second-order $\delta\Delta^{(2)}$ sideband inequivalences take on similar expansions as

$$\frac{\delta\Delta^{(1)}}{\Omega} \approx -\frac{\delta\Delta^{(2)}}{2\Omega} \approx \left(\frac{g_0}{\Omega}\right)^2 \left(\bar{n}+\frac{1}{2}\right) - 2\left(\frac{g_0}{\Omega}\right)^4 \left(\bar{n}+\frac{1}{2}\right)\left(\bar{m}+\frac{1}{2}\right) \approx \mathscr{G}^2 - 4\mathscr{G}^4\mathscr{G}_0^2. \tag{S54}$$

Here, $\mathscr{G}_0 = g_0/\Omega$ and $\mathscr{G} = g/\Omega$ are normalized interaction rates with respect to the mechanical frequency, where $g = g_0\sqrt{\bar{n}}$ is the enhanced optomechanical interaction rate. Furthermore, $\bar{m}$ is approximated from (S30) in the above.

Results of noise spectrum calculations using the fully linearized and higher-order formulations of optomechanics is shown in Fig. S9. The single-photon optomechanical interaction rate $g_0 = 1.68 \times 10^{-3}\Omega$ and the enhanced optomechanical interaction on resonance satisfies $g = 0.31\Omega$, corresponding to the strong coupling regime and optical power of $P_{\mathrm{op}} = 47.2\mu\mathrm{W}$ at $T = 3\mathrm{K}$. Cavity is side-band resolved with the parameters given elsewhere [S15, S18]. The input power is high-enough to cause the cavity to exhibit asymmetric reflectivity, a very clear hallmark of bistability seen easily in experiments.

This has been calculated and illustrated in Fig. S10 for various linear and higher-order formulations resulting from simulating a scanning pump experiment. It can be seen that the fully-linearized optomechanics cannot reasonably reproduce the highly asymmetric and non-Lorentzian lineshape of cavity under strong pump. As a simple measure of reflectivity, one may use the Langevin equation for photons $\hat{a}$ with the semi-classical substitutions $\hat{a} \to \bar{a}$ and $\hat{b} \to \bar{b}$, where $\bar{b}$ is correspondingly

given from (S6), and $\bar{n}$ can be nonlinearly solved from (S9). For a side-coupled cavity where the reflectivity is not identity, and external coupling rate κ_{ex} is known, we have $\eta = \kappa_{ex}/\kappa$, leading to the approximation

$$\mathscr{R}(\omega,\Delta) = 1 - \frac{i\kappa_{ex}}{\omega+\Delta+2g_0^2\bar{n}\frac{\Omega}{\Omega^2+\frac{1}{4}\Gamma^2}+i\frac{1}{2}\kappa}, \tag{S55}$$

where $|\mathscr{R}|^2$ is plotted as the dot-dashed curve in Fig. S10. More accurate solutions can be found by taking the scattering matrix element $\mathscr{R} = Y_{11}$, also shown in Fig. S10.

The existence of tiny second-order sidebands around $\pm 2\Omega$ is illustrated by calculation of the reflectivity near the corresponding resonances. This has been shown in Fig. S11 for both of the second-order red and blue sidebands at $P_{op} = 3$mW. The depths of these resonances are rather small, being only around -0.13mdB.

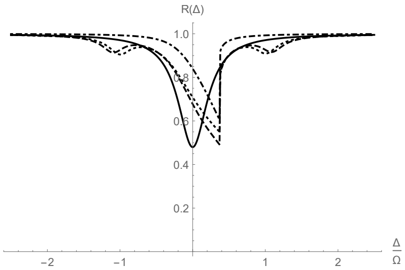

Figure S10. Estimated reflectivity $|Y_{11}(\omega)|^2$ of a side-band resolved optomechanical cavity under single pump with varying frequency according to the fully linearized optomechanics in solid black, higher-order optomechanics with the bases $\{A\}^{\mathrm{T}} = \left\{\hat{a}, \hat{a}\hat{b}, \hat{a}\hat{b}^\dagger\right\}$ and (S52) respectively shown in dashed and dotted curves, and approximate semi-classical calculation using (S6), $\hat{b} \to \bar{b}$, $\hat{a} \to \bar{a}$ and the Langevin equation for $\hat{a}$.

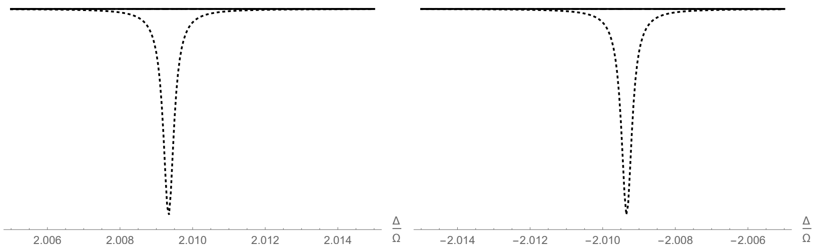

Figure S11. Illustration of tiny second-order sidebands around $\pm 2\Omega$ by calculation of reflectivity $|Y_{11}(\omega)|^2$ from a side-band resolved optomechanical cavity under single pump with varying frequency. Only the higher-order optomechanics with the bases (S52) shown in dotted curve may exhibit non-trivial behavior near second-order side-bands: second-order red sideband (left); second-order blue sideband (right).

S10 Multiplicative Noise

Rewriting (S53) without the zeroth-order approximation for multplicative noise, gives the exact higher-order set of Langevin equations

$$\frac{d}{dt}\left\{\begin{array}{c}\hat{a}\\ \hat{a}\hat{b}\\ \hat{a}\hat{b}^\dagger\\ \hat{a}\hat{b}^2\\ \hat{a}\hat{b}^{\dagger 2}\end{array}\right\} = \begin{bmatrix} i\Delta-\frac{\kappa}{2} & ig_0 & ig_0 & 0 & 0\\ ig_0(\bar{m}+\bar{n}+1) & -i(\Omega-\Delta)-\frac{\gamma}{2} & 0 & ig_0 & 0\\ ig_0(\bar{m}-\bar{n}) & 0 & i(\Omega+\Delta)-\frac{\gamma}{2} & 0 & ig_0\\ 0 & ig_0(\bar{m}+2\bar{n}+2) & 0 & -i(2\Omega-\Delta)-\frac{\theta}{2} & 0\\ 0 & 0 & ig_0(\bar{m}-2\bar{n}-1) & 0 & i(2\Omega+\Delta)-\frac{\theta}{2}\end{bmatrix}\left\{\begin{array}{c}\hat{a}\\ \hat{a}\hat{b}\\ \hat{a}\hat{b}^\dagger\\ \hat{a}\hat{b}^2\\ \hat{a}\hat{b}^{\dagger 2}\end{array}\right\}$$
$$-\begin{bmatrix}\sqrt{\kappa} & 0 & 0\\ \sqrt{\kappa}\hat{b} & \sqrt{\Gamma}\hat{a} & 0\\ \sqrt{\kappa}\hat{b}^\dagger & 0 & \sqrt{\Gamma}\hat{a}\\ \sqrt{\kappa}\hat{b}^2 & \sqrt{\Gamma}\hat{a}\hat{b} & 0\\ \sqrt{\kappa}\hat{b}^{\dagger 2} & 0 & \sqrt{\Gamma}\hat{a}\hat{b}^\dagger\end{bmatrix}\left\{\begin{array}{c}\hat{a}_{in}\\ \hat{b}_{in}\\ \hat{b}_{in}^\dagger\end{array}\right\} + \begin{bmatrix}1 & 0\\ \bar{b} & 0\\ \bar{b}^* & 0\\ \bar{b}^2 & 0\\ \bar{b}^{*2} & 0\end{bmatrix}\left\{\begin{array}{c}\alpha\\ \alpha^*\end{array}\right\}. \tag{S56}$$

To illustrate how the multiplicative noise terms on the second line are to be treated, let us assume that a simple equation is given as

$$\frac{d}{dt}\mathscr{A}(t)=(i\Delta-\frac{1}{2}\kappa)\mathscr{A}(t)-\sqrt{\kappa}\hat{x}(t)\hat{a}_{\text{in}}(t), \tag{S57}$$

where $\hat{x}$ is some dimensionless and time-dependent operator and $\hat{a}_{\text{in}}$ corresponds to a white noise random process. The spectral density of the zero-average operator $\mathscr{A}$ by definition is

$$S_{\mathscr{A}\mathscr{A}}(w)=\int_{-\infty}^{\infty}d\tau e^{iw\tau}\left\langle\mathscr{A}^{\dagger}(t)\mathscr{A}(t+\tau)\right\rangle. \tag{S58}$$

Therefore, the symmetrized spectral density via symmetrization operator $\mathscr{S}$ which is the actual quantity measured in experiments is

$$\begin{aligned}\mathscr{S}S_{\mathscr{A}\mathscr{A}}(w) &= \int_{-\infty}^{\infty}d\tau e^{iw\tau}\left\langle\mathscr{S}\{\mathscr{A}^{\dagger}(t)\mathscr{A}(t+\tau)\}\right\rangle \\ &= \int_{-\infty}^{\infty}d\tau e^{iw\tau}\left\langle\mathscr{A}^{\dagger}(t)\mathscr{A}(t+\tau)\right\rangle_{\text{S}}.\end{aligned} \tag{S59}$$

The equation (S57) admits a formal solution

$$\mathscr{A}(t)=-\sqrt{\kappa}\mathbb{L}(t)\hat{x}(t)\hat{a}_{\text{in}}(t), \tag{S60}$$

where $\mathbb{L}$ is given as

$$\mathbb{L}(t)=\left(\frac{d}{dt}-i\Delta+\frac{1}{2}\kappa\right)^{-1}, \tag{S61}$$

and is an operator which can be understood as an inverse Fourier transform such as

$$\mathbb{L}(t)=\mathscr{F}^{-1}\left(\frac{1}{iw-i\Delta+\frac{1}{2}\kappa}\right)(t)=\mathscr{F}^{-1}\{L(w)\}(t). \tag{S62}$$

Here, we are not interested in an explicit form of $\mathbb{L}$ although it is easy to be evaluated or looked up from table of Fourier transforms.

The formal solution (S60) gives rise to the symmetrized spectral density

$$\mathscr{S}S_{\mathscr{A}\mathscr{A}}(w)=\kappa\int_{-\infty}^{\infty}d\tau e^{iw\tau}\left\langle\hat{a}_{\text{in}}^{\dagger}(t)\hat{x}^{\dagger}(t)\mathbb{L}^{\dagger}(t)\mathbb{L}(t+\tau)\hat{x}(t+\tau)\hat{a}_{\text{in}}(t+\tau)\right\rangle_{\text{S}}. \tag{S63}$$

Now, we can employ the Isserlis-Wick theorem to decompose the expectation value as [S3, S19–S21]

$$\begin{aligned}\left\langle\hat{a}_{\text{in}}^{\dagger}(t)\hat{y}^{\dagger}(t)\hat{y}(t+\tau)\hat{a}_{\text{in}}(t+\tau)\right\rangle_{\text{S}} &= \left\langle\hat{a}_{\text{in}}^{\dagger}(t)\hat{a}_{\text{in}}(t+\tau)\right\rangle_{\text{S}}\left\langle\hat{y}^{\dagger}(t)\hat{y}(t+\tau)\right\rangle_{\text{S}} \\ &+ \left\langle\hat{a}_{\text{in}}^{\dagger}(t)\hat{y}^{\dagger}(t)\right\rangle_{\text{S}}\left\langle\hat{y}(t+\tau)\hat{a}_{\text{in}}(t+\tau)\right\rangle_{\text{S}} \\ &+ \left\langle\hat{a}_{\text{in}}^{\dagger}(t)\hat{y}(t+\tau)\right\rangle_{\text{S}}\left\langle\hat{y}^{\dagger}(t)\hat{a}_{\text{in}}(t+\tau)\right\rangle_{\text{S}}.\end{aligned} \tag{S64}$$

where $\hat{y}(t)=\mathbb{L}(t)\hat{x}(t)$ is adopted for shorthand notation. Since $\hat{a}_{\text{in}}$ is a white noise Wiener random process, we may expect that to a very good approximation the second and third terms vanish and thus

$$\mathscr{S}S_{\mathscr{A}\mathscr{A}}(w)=\kappa\int_{-\infty}^{\infty}d\tau e^{iw\tau}\left\langle\hat{a}_{\text{in}}^{\dagger}(t)\hat{a}_{\text{in}}(t+\tau)\right\rangle_{\text{S}}\left\langle\hat{y}^{\dagger}(t)\hat{y}(t+\tau)\right\rangle_{\text{S}}. \tag{S65}$$

The random nature of a Weiner process requires that [S1]

$$\begin{aligned}\mathscr{S}S_{\mathscr{A}\mathscr{A}}(w) &= \left|\kappa\int_{-\infty}^{\infty}d\tau e^{iw\tau}\left\langle\hat{y}^{\dagger}(t)\hat{y}(t+\tau)\right\rangle_{\text{S}}\right|^{2}\mathscr{S}S_{AA}(w) \\ &= |L(w)*\hat{x}(w)|^{2}\mathscr{S}S_{AA}(w).\end{aligned} \tag{S66}$$

Here, $*$ represents convolution in frequency and $L(w)$ is defined in (S62) and actually represents the equivalent to the scattering matrix element. The expression (S66) presents a mathematically exact solution to the spectral density problem of multiplicative noise (S57).

In the context of higher-order quantum optomechanics and referring to (S56) the operator $\hat{x}$ may represent either of the operators within the set $\{\hat{a},\hat{b},\hat{b}^\dagger,\hat{a}\hat{b},\hat{a}\hat{b}^\dagger,\hat{b}^2,\hat{b}^{\dagger 2}\}$. However, not only these are not yet known, but also, they are influenced by random processes from the correspondingly lower-order interactions with the optical field and mechanical bath. The only approximation needed here is to replace these with corresponding non-operator functions which can be already obtained from the solution to lower-order equations. Doing this results in a set of equations for $\{\bar{a},\bar{b},\bar{b}^*,\overline{ab},\overline{ab^*},\bar{b}^2,\bar{b}^{*2}\}$, where solutions for $\{\bar{b},\bar{b}^*,\bar{b}^2,\bar{b}^{*2}\}$ can be obtained by having $\bar{b}$. This is here calculated from the 3×3 linearized optomechanics, giving rise to the expression

$$\bar{b}(w)=\frac{\alpha}{i(w+\Omega)+\frac{1}{2}\Gamma}. \tag{S67}$$

In a similar manner to §S6, the next required expressions can be explicitly obtained by Mathematica as

$$\begin{aligned}
\bar{a}(w) &= \frac{-\alpha^2 g_0(w-\Delta-\Omega-i\frac{1}{2}\gamma)}{(w+\Omega-\frac{1}{2}i\Gamma)\left\{2g_0^2\left[(\Delta-w+\frac{1}{2}i\gamma)(\bar{m}+\frac{1}{2})+\Omega(\bar{n}+\frac{1}{2})\right]+(w-\Delta-\frac{1}{2}i\kappa)\left[\left(w-\Delta-\frac{1}{2}i\gamma\right)^2-\Omega^2\right]\right\}}, \\
\overline{ab}(w) &= \frac{\alpha^2\left[g_0^2(\bar{m}-\bar{n})-(w-\Delta-\frac{1}{2}i\kappa)(w-\Delta-\Omega-\frac{1}{2}i\gamma)\right]}{(w+\Omega-\frac{1}{2}i\Gamma)\left\{2g_0^2\left[(\Delta-w+\frac{1}{2}i\gamma)(\bar{m}+\frac{1}{2})+\Omega(\bar{n}+\frac{1}{2})\right]+(w-\Delta-\frac{1}{2}i\kappa)\left[\left(w-\Delta-\frac{1}{2}i\gamma\right)^2-\Omega^2\right]\right\}}, \\
\overline{ab^*}(w) &= \frac{-|\alpha|^2\left[g_0^2(\bar{m}+\bar{n}+1)-(w-\Delta-\frac{1}{2}i\kappa)(w-\Delta+\Omega-\frac{1}{2}i\gamma)\right]}{(w+\Omega+\frac{1}{2}i\Gamma)\left\{2g_0^2\left[(\Delta-w+\frac{1}{2}i\gamma)(\bar{m}+\frac{1}{2})+\Omega(\bar{n}+\frac{1}{2})\right]+(w-\Delta-\frac{1}{2}i\kappa)\left[\left(w-\Delta-\frac{1}{2}i\gamma\right)^2-\Omega^2\right]\right\}}.
\end{aligned} \tag{S68}$$

In the above equations, it has to be noticed that α is a complex quantity which satisfies $|\alpha|=\sqrt{\kappa_{\rm ex}P_{\rm op}/\hbar\omega}$, and also by (S8) we have

$$\alpha=\sqrt{\bar{n}}\left[-\frac{\kappa}{2}+i\left(\Delta+\frac{2g_0^2\Omega}{\Omega^2+\frac{1}{4}\Gamma^2}\bar{n}\right)\right]. \tag{S69}$$

Now, we can rewrite the Langevin equations (S56) as

$$\begin{aligned}
\frac{d}{dt}\{\delta A\} &= [\mathbf{M}]\{\delta A\}-[\hat{\mathbf{G}}]\{A_{\rm in}\}, \\
[\mathbf{M}] &= \begin{bmatrix} i\Delta-\frac{\kappa}{2} & ig_0 & ig_0 & 0 & 0 \\ ig_0(\bar{m}+\bar{n}+1) & -i(\Omega-\Delta)-\frac{\gamma}{2} & 0 & ig_0 & 0 \\ ig_0(\bar{m}-\bar{n}) & 0 & i(\Omega+\Delta)-\frac{\gamma}{2} & 0 & ig_0 \\ 0 & ig_0(\bar{m}+2\bar{n}+2) & 0 & -i(2\Omega-\Delta)-\frac{\theta}{2} & 0 \\ 0 & 0 & ig_0(\bar{m}-2\bar{n}-1) & 0 & i(2\Omega+\Delta)-\frac{\theta}{2} \end{bmatrix}, \\
\{\delta A\}^{\rm T} &= \left\{\delta\hat{a},\delta(\hat{a}\hat{b}),\delta(\hat{a}\hat{b}^\dagger),\delta(\hat{a}\hat{b}^2),\delta(\hat{a}\hat{b}^{\dagger 2})\right\}, \\
\{A_{\rm in}\}^{\rm T} &= \left\{\hat{a}_{\rm in},\hat{b}_{\rm in},\hat{b}_{\rm in}^\dagger\right\}, \\
[\hat{\mathbf{G}}] &= \begin{bmatrix} \sqrt{\kappa} & 0 & 0 \\ \sqrt{\kappa}\hat{b} & \sqrt{\Gamma}\hat{a} & 0 \\ \sqrt{\kappa}\hat{b}^\dagger & 0 & \sqrt{\Gamma}\hat{a} \\ \sqrt{\kappa}\hat{b}^2 & \sqrt{\Gamma}\hat{a}\hat{b} & 0 \\ \sqrt{\kappa}\hat{b}^{\dagger 2} & 0 & \sqrt{\Gamma}\hat{a}\hat{b}^\dagger \end{bmatrix}.
\end{aligned} \tag{S70}$$

After defining the decay matrix

$$[\sqrt{\Gamma}]=\begin{bmatrix} \sqrt{\kappa} & 0 & 0 & 0 & 0 \\ 0 & \sqrt{\gamma} & 0 & 0 & 0 \\ 0 & 0 & \sqrt{\gamma} & 0 & 0 \\ 0 & 0 & 0 & \sqrt{\theta} & 0 \\ 0 & 0 & 0 & 0 & \sqrt{\theta} \end{bmatrix}, \tag{S71}$$

taking the Fourier transform, and using the input-output relation

$$\{A_{\rm out}(\omega)\}=\{A_{\rm in}(\omega)\}+[\sqrt{\Gamma}]^{\rm T}\{\delta A(\omega)\}, \tag{S72}$$

we arrive at the definition of the scattering matrix

$$[\mathbf{Y}(\omega)]=\mathbf{I}-[\sqrt{\Gamma}]^{\mathrm{T}}\left([\mathbf{M}]-i\omega[\mathbf{I}]\right)^{-1}[\sqrt{\Gamma}], \tag{S73}$$

by which and (S66) we can evaluate the desired symmetrized spectral density of output optical field as

$$\begin{aligned} S(\omega) &= |Y_{11}(\omega)|^2 S_{AA}(\omega) \\ &+ \frac{1}{\gamma^2}\left|[Y_{12}(\omega)+Y_{13}(\omega)]*\bar{a}(\omega)\right|^2 S_{BB}(\omega) \\ &+ \frac{1}{\theta^2}\left|\left[Y_{14}(\omega)*\overline{ab}(\omega)+Y_{15}(\omega)*\overline{ab^*}(\omega)\right]\right|^2 S_{BB}(\omega), \end{aligned} \tag{S74}$$

where spectral power densities S_{AA} and S_{BB} are already introduced in (S11) and convolutions $*$ take place over the entire frequency axis.

S11 Elements of Higher-order Scattering Matrices

This section reports the explicit elements of the first row of scattering matrix $[\mathbf{Y}]$ in (S73), as needed for calculation of the spectral density according to (S74). These might be useful only when the method of residues are to be used for exact evaluation of complex convolution integrals, otherwise full numerical simulation of (S74) is much preferable.

S11.1 Second-order 3×3 Formalism

The elements of the scattering matrix are explicitly found using the supplementary Mathematica packages, and after some simplification they take the form

$$\begin{aligned} Y_{11}(\omega) &= 1-\frac{2i\kappa_{\mathrm{ex}}\left[\left(\omega-\Delta-\frac{1}{2}i\gamma\right)^2-\Omega^2\right]}{2g_0^2\left[(\Delta-\omega+\frac{1}{2}i\gamma)(\bar{m}+\frac{1}{2})+\Omega(\bar{n}+\frac{1}{2})\right]+(\omega-\Delta-\frac{1}{2}i\kappa)\left[\left(\omega-\Delta-\frac{1}{2}i\gamma\right)^2-\Omega^2\right]}, \\ Y_{12}(\omega) &= \frac{-ig_0\sqrt{\gamma\kappa_{\mathrm{ex}}}\left(\omega-\Delta-\Omega-\frac{1}{2}i\gamma\right)}{2g_0^2\left[(\Delta-\omega+\frac{1}{2}i\gamma)(\bar{m}+\frac{1}{2})+\Omega(\bar{n}+\frac{1}{2})\right]+(\omega-\Delta-\frac{1}{2}i\kappa)\left[\left(\omega-\Delta-\frac{1}{2}i\gamma\right)^2-\Omega^2\right]}, \\ Y_{13}(\omega) &= \frac{-ig_0\sqrt{\gamma\kappa_{\mathrm{ex}}}\left(\omega-\Delta+\Omega-\frac{1}{2}i\gamma\right)}{2g_0^2\left[(\Delta-\omega+\frac{1}{2}i\gamma)(\bar{m}+\frac{1}{2})+\Omega(\bar{n}+\frac{1}{2})\right]+(\omega-\Delta-\frac{1}{2}i\kappa)\left[\left(\omega-\Delta-\frac{1}{2}i\gamma\right)^2-\Omega^2\right]}. \end{aligned} \tag{S75}$$

These expressions are useful in speed up of the code, as well as wherever the method of residues is to be used.

S11.2 Third-order 5×5 Formalism

The convergence of 3×3 is sufficiently good for most practical purposes, and also the explicit expressions for 5×5 matrices decompose into products of fourth-order polynomials in terms of ω in their denominators, which severely limits the usefulness of applicability of the method of residues. For this reason, their explicit expressions are not included here. The interested reader may find them in the supplementary Mathematica packages instead.

Mathematica Packages

These are brief descriptions of supplied Mathematica packages along with this article, written by the author:

- SuppleMath1.nb: Derivation of coherent phonon population $\bar{m}(\Delta)$ in S30.
- SuppleMath2.nb: Derivation of expressions corresponding to §S11.1 and §S11.2.
- SuppleMath3.nb: Code for generation of noise spectra in the main article including animated graphs.
- SuppleMath4.nb: Code for generation of stability diagrams in the main article.
- SuppleMath5.nb: Code for generation of higher-order optical spring effect in Fig. S6.
- SuppleMath6.nb: Code for generation of higher-order optical spring effect in Fig. S7.
- SuppleMath7.nb: Code for generation of higher-order optical spring effect in Fig. S8.
- SuppleMath8.nb: Numerical calculation of side-band inequivalence.
- SuppleMath9.nb: Code for generation of normalized side-band inequivalence in of the main article.
- SuppleMath10.nb: Code for generation of reflection spectra in Fig. S10.
- SuppleMath11.nb: Code for generation of second-order side-bands in Fig. S11.

References

[S1] Bowen, W. P. & Milburn, G. J. *Quantum Optomechanics* (CRC Press: Boca Raton, 2016).

[S2] Aspelmeyer, M, Kippenberg, T. J. & Marquardt, F. Cavity optomechanics. *Rev. Mod. Phys.* **86**, 1391 (2014).

[S3] Khorasani, S. Higher-order interactions in quantum optomechanics: Analytical solution of nonlinearity. *Photonics* **4**, 48 (2017).

[S4] Kippenberg, T. J. & Vahala, K. J. Cavity optomechanics. *Science* **321**, 1172 (2008).

[S5] Doolin, C., Hauer, B. D., Kim, P. H., MacDonald, A. J. R., Ramp, H. & Davis, J. P. Nonlinear optomechanics in the stationary regime. *Phys. Rev. A* **89**, 053838 (2014).

[S6] Teufel, J. D., Harlow, J. W., Regal, C. A. & Lehnert, K. W. Dynamical backaction of microwave fields on a nanomechanical oscillator. *Phys. Rev. Lett.* **101**, 197203 (2008).

[S7] Eichenfield, M., Camacho, R., Chan, J., Vahala, K. J. & Painter, O. A picogram- and nanometre-scale photonic-crystal optomechanical cavity. *Nature* **459**, 550 (2009).

[S8] Safavi-Naeini, A. H., Gröblacher, S., Hill, J. T., Chan, J., Aspelmeyer, M. & Painter, O. Squeezed light from a silicon micromechanical resonator. *Nature* **500**, 185 (2013).

[S9] Deotare, P. B., Bulu, I., Frank, I. W., Quan, Q., Zhang, Y., Ilic, R. & Loncar, M. All optical reconfiuration of optomechanical fiters. *Nat. Commun.* **3**, 846 (2011).

[S10] Sarabalis, C. J., Dahmani, Y. D., Patel, R. N., Hill, J. T. & Safavi-Naeini, A. H.. Release-free silicon-on-insulator cavity optomechanics. *Optica* **4**, 1147 (2017).

[S11] Hong, S., Riedinger, R., Marinkovic, I., Wallucks, A., Hofer, S. G., Norte, R. A., Aspelmeyer, M. & Gröblacher, S. Hanbury Brown and Twiss interferometry of single phonons from an optomechanical resonator. *Science* **358**, 203 (2017).

[S12] Riedinger, R., Wallucks, A., Marinkovic, I., Löschnauer, C., Aspelmeyer, M., Hong, S. & Gröblacher, S. Remote quantum entanglement between two micromechanical oscillators. *Nature* **556**, 473 (2018).

[S13] Macrì, V., Ridolfo, A., Di Stefano, O., Kockum, A. F., Nori, F. & Savasta, S. Non-perturbative dynamical Casimir effect in optomechanical systems: Vacuum Casimir-Rabi splittings. *Phys. Rev. X* **8**, 011031 (2018).

[S14] Leijssen, R. & Verhagen, E. Strong optomechanical interactions in a sliced photonic crystal nanobeam. *Sci. Rep.* **5**, 15974 (2015).

[S15] Schliesser, A. & Kippenberg, T. J. Cavity optomechanics with whispering-gallery-mode optical micro-resonators. *Adv. At., Mol. Opt. Phys.* **58**, 207 (2010).

[S16] Loo, S. G. Spectral density of random signals corrupted by multiplicative noise. *Electron. Lett.* **3**, 238 (1967).

[S17] Børkje, K., Nunnenkamp, A., Teufel, J. D. & Girvin, S. M. Signatures of nonlinear cavity optomechanics in the weak coupling regime. *Phys. Rev. Lett.* **111**, 053603 (2012).

[S18] Schliesser, A., Rivière, R., Anetsberger, G., Arcizet, O. & Kippenberg, T. J. Resolved-sideband cooling of a micromechanical oscillator. *Nature Phys.* **4**, 415 (2008).

[S19] Hübener, R., Mari, A. & Eisert, J. Wick's theorem for matrix product states. *Phys. Rev. Lett.* **110**, 040401 (2013).

[S20] Isserlis, L. On a formula for the product-moment coefficient of any order of a normal frequency distribution in any number of variables. *Biometrika* **12**, 134 (1918).

[S21] Wick, G.C. The evaluation of the collision matrix. *Phys. Rev.* **80**, 268 (1950).
